# Integrated study on the occurrence and genomic features of *Escherichia albertii* in environmental water and raccoons in Japan

**DOI:** 10.1128/aem.00076-26

**Published:** 2026-03-24

**Authors:** Atsushi Hinenoya, Rin Tagami, Sharda Prasad Awasthi, Bingting Xu, Noritoshi Hatanaka, Shinji Yamasaki

**Affiliations:** 1 School of Life and Environmental Sciences, Osaka Prefecture University, Osaka, Japan; 2 Graduate School of Veterinary Science, Osaka Metropolitan University 12936, Osaka, Japan; 3 Asian Health Science Research Institute, Osaka Metropolitan University 12936, Osaka, Japan; 4 Osaka International Research Center for Infectious Diseases, Osaka Metropolitan University, Osaka, Japan; 5 Graduate School of Life and Environmental Sciences, Osaka Prefecture University, Osaka, Japan; Anses, Maisons-Alfort Laboratory for Food Safety, Maisons-Alfort, France

**Keywords:** raccoon, environmental water, *Escherichia albertii*

## Abstract

*Escherichia albertii* is an emerging zoonotic pathogen that causes intestinal diseases in humans and has been linked to several outbreaks, particularly in Japan. To better understand its environmental ecology and transmission dynamics, we conducted integrated surveillance of environmental water and wild raccoons in Osaka, Japan. *E. albertii* was frequently detected in river water (76.6%) and raccoons (57.7%) using species-specific PCR. Sequential water sampling at two river locations, performed three times at 30-min intervals, consistently yielded PCR-positive results, suggesting that *E. albertii* could be detected repeatedly at the same sites despite ongoing water flow. Further analysis indicated the continued detectability of certain *E. albertii* clones together with additional genotypes. Whole-genome sequencing of 147 strains found that all strains harbored virulence genes associated with clinical strains, along with substantial genomic diversity, overlapping phylogeny, and clonal relationships between strains from both sources. Notably, one raccoon strain and one water strain, collected in the same city and period, differed by fewer than 20 single-nucleotide polymorphisms and showed only a single-band difference in pulsed-field gel electrophoresis, suggesting possible inter-source transmission. Moreover, some environmental and raccoon strains were closely related to human clinical strains, including one linked to a large outbreak in 2017. These findings suggest that *E. albertii* is widely distributed in aquatic environments and wildlife in Japan and has the potential to cause human infections. This study provides new insights into the ecology and public health risks of *E. albertii*, highlighting the importance of a One Health approach to monitor this emerging pathogen.

**IMPORTANCE:**

Understanding how zoonotic pathogens circulate in the environment is essential for preventing emerging infectious diseases. *E. albertii* is increasingly recognized as a clinically relevant, emerging foodborne pathogen with global distribution. However, its ecological reservoirs, environmental persistence, and transmission dynamics remain poorly understood. This study is the first report to demonstrate a high prevalence and repeated detectability of *E. albertii* in environmental water and to show its genetic links among strains isolated from environmental water, wild raccoons, and human clinical cases. These findings improve our understanding of the environmental ecology of *E. albertii* and suggest its potential for transmission across environmental, animal, and human interfaces. The results underscore the importance of a One Health-based surveillance strategy to effectively monitor and control this pathogen. Furthermore, this work provides a basis for future investigations into environmental sources of bacterial zoonotic agents.

## INTRODUCTION

*Escherichia albertii* is a recently recognized zoonotic foodborne pathogen that causes diarrhea, abdominal pain, vomiting, and fever in humans (1–3). *E. albertii* is a Gram-negative, facultative anaerobic, non-spore-forming, rod-shaped bacterium that exhibits no motility under routine biochemical examination. This bacterium is closely related to *Escherichia coli* and shares hallmark virulence factors with diarrheagenic *E. coli*, including enteropathogenic *E. coli* (EPEC) and enterohemorrhagic *E. coli* (EHEC). Notably, *E. albertii* carries a pathogenicity island known as the locus of enterocyte effacement (LEE) encoding the type three secretion system (T3SS) and *eae* gene-encoded adhesin, intimin. *E. albertii* also frequently harbors an ortholog of the *paa* gene encoding Paa (porcine attaching and effacing associated) (4), originally identified in *E. coli* and might be associated with the early stages of bacterial adherence (5).

*E. albertii* produces cytolethal distending toxin (CDT) encoded by the *Eacdt* gene (almost ubiquitously detected) and the *Eccdt-I* gene (an ortholog of *cdt-I* genes in *E. coli*; less frequently detected), which could be associated with virulence and persistent colonization by CDT-producing bacteria (6). Certain strains produce Shiga toxin 2 (Stx2a or Stx2f) (7–9), a primary virulence factor of EHEC. Because of the similar phenotypic and genotypic characteristics to other bacteria belonging to Enterobacterales, *E. albertii* has often been misidentified as other bacterial species, such as *Hafnia alvei*, *Shigella boydii*, EPEC, EHEC, and CDT-II-producing *E. coli* (1, 2, 10–13), suggesting that *E. albertii* infections in humans have been underestimated. Recently, this bacterium has been responsible for several outbreaks of food poisoning, particularly in Japan and in a recent case in China (13–15), and the clinical importance of this pathogen has been increasingly recognized worldwide.

Many epidemiological studies have been done to explore the *E. albertii* animal reservoirs and potential human infection routes. Wildlife surveys detected this bacterium in various mammals, such as raccoons, raccoon dogs, badgers, martens, masked palm civets, boars, foxes, bats, dogs, and cats (13, 16–19), suggesting that they may serve as the reservoirs or carriers. Notably, raccoons are considered one of the natural reservoirs of this bacterium (3, 17, 18). Reports from several countries have also shown wild birds carrying this bacterium (20–23). Among livestock, *E. albertii* has been detected frequently in chickens (2, 16, 24–26) and their meats with a higher occurrence than other meats (beef, mutton, and pork) (26–30), suggesting that poultry may be an important source for human infections.

The presence of *E. albertii* in environmental water, such as rivers, moats, and municipal drains, has been increasingly reported (18, 31, 32). The *E. albertii* strains isolated from these sources harbor virulence genes, and some exhibit O-antigen genotypes that have also been identified in human clinical strains (33). Environmental water is used to irrigate crops and vegetables, and *E. albertii* has been detected in vegetables, including kale, lettuce, and spinach (34, 35). Notably, some outbreaks in Japan have been linked to spring water, well water, and vegetables, suggesting that water and produce could also be sources of human infection (14). Wild animals, such as raccoons, might contribute to the contamination of environmental water and agricultural products. However, significant knowledge gaps remain regarding the prevalence of *E. albertii* in environmental water and the potential role of wild animals in its transmission.

In this study, we conducted integrated surveillance of environmental water and wild raccoons in Osaka, Japan, to investigate the presence of *E. albertii*. The *E. albertii* strains isolated were comparatively analyzed using whole-genome sequencing and compared to newly sequenced human clinical strains from Japan. The novel findings of this study provide critical insights into the ecology and transmission dynamics of *E. albertii* and underscore the need to control the emerging *E. albertii* within a complex ecosystem through a One Health approach.

## RESULTS

### Occurrence of *E. albertii* in environmental water

Environmental water, with a focus on rivers, was sampled in Osaka, Japan, with a total of 64 samples collected from eight riverine systems (Table 1; Table S1). Based on the hypothesis that environmental water may be contaminated with *E. albertii* by wild animals, samples were collected between 2022 and 2023 from the upper and middle reaches of rivers that overlap with wildlife habitat areas. *E. albertii* was detected in 49 out of the 64 water samples (76.6%) using an *E. albertii*-specific real-time PCR (Table 1), corresponding to 6 out of 8 riverine systems testing PCR-positive (75.0%) (Table 1).

**TABLE 1:** Overall detection, isolation, and PCR-typing of *Escherichia albertii* from environmental water and wild raccoons^*a*^

Sampling source	Riverine system	No. of samples			No. of strains	No. of ERIC patterns			
		Tested	PCR positive (%)	Isolation positive		1	2	3	4
Water	1	26	23 (88.5%)	17	31	4	12	1	0
	2	17	15 (88.2%)	15	36	2	8	4	1
	3	6	4 (66.7%)	3	5	0	2	1	0
	4	4	2 (50.0%)	1	1	1	0	0	0
	5	6	3 (50.0%)	2	5	1	0	0	1
	6	2	2 (100%)	1	1	1	0	0	0
	7	2	0 (0%)	NA	NA	NA	NA	NA	NA
	8	1	0 (0%)	NA	NA	NA	NA	NA	NA
	Total	64	49 (76.6%)	39	79	9	22	6	2
Raccoon		122	68 (55.7%)	56	68	44	12	0	0

^*a*^ NA, not applicable.

**Fig 1:**
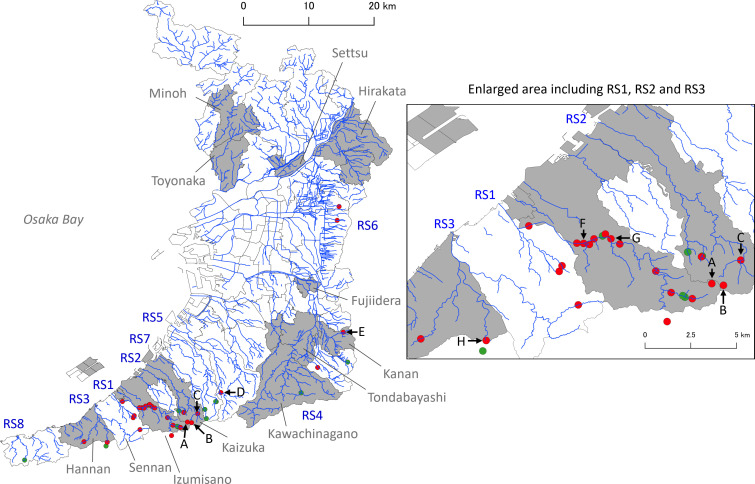
Map showing the sampling locations of environmental water and the cities and towns where *Escherichia albertii* PCR-positive raccoons were captured in Osaka, Japan. Environmental water samples were collected from rivers and one spring belonging to eight riverine systems (RS1–RS8). Red dots indicate *E. albertii* PCR-positive locations, and green dots indicate PCR-negative locations. The cities and towns where *E. albertii*-positive raccoons were captured in this study are shaded in gray. An enlarged view of the area covering RS1, RS2, and RS3 is provided. Highlighted sampling locations are alphabetically labeled (A–H). The original map of Osaka was obtained from the website of the Geospatial Information Authority of Japan and modified using QGIS software.

From riverine system 1, water samples were extensively collected from the middle to upper reaches, including tributaries (Fig. 1). Fifteen out of 18 sites (83.3%) tested PCR-positive, and even the water sample collected near the river source (EW2208; location A in Fig. 1; Table S1) was positive. Similarly, water samples from the upper reaches of other riverine systems, such as EW2210 (location B), EW2305 (location C), EW2315 (location D), and EW2319 (location E), were also PCR-positive.

Water samples were repeatedly collected from three selected locations (Table S1). At the first location (location F in Fig. 1), samplings were conducted twice in 2022 (samples EW2201 and EW2212; August 16 and October 17) and twice in 2023 (EW2323 and EW2334; April 3 and June 15). Similarly, at the second location (location G), samples were collected twice in 2022 (EW2205 and EW2221; September 13 and December 17) and twice in 2023 (EW2330 and EW2347; May 12 and October 7). At the third location (location H), samples were collected once in 2022 (EW2213; October 17) and three times in 2023 (EW2302, EW2322, and EW2333; January 9, April 3, and June 15). Interestingly, all samples collected from these three locations, except for sample EW2322 from the third location on April 3, were consistently PCR-positive for *E. albertii*.

Additionally, at two locations, water samples were collected repeatedly to assess short-term and temporal detectability of *E. albertii*. At one location (location G in Fig. 1), three samples were collected at 30-min intervals on May 12, 2023 (samples EW2330 to EW2332; Table S1) to evaluate short-term detectability. At another location (location B), samples were collected on multiple dates (July 3, July 31, August 7, and September 5, in 2023; samples EW2335 to EW2346; Table S1) to assess detectability in short-term and longer time periods. All sequentially collected samples were positive for *E. albertii* by PCR. These results indicate that *E. albertii* was repeatedly detectable at the same sampling locations over short time intervals as well as across different sampling dates, despite ongoing water flow. This suggests that the water samples collected throughout this study reliably reflected the presence of *E. albertii* contamination at the time of sampling in the surveyed river waters.

Out of 49 PCR-positive samples, *E. albertii* was successfully isolated from 39 samples using a selective isolation medium (Table 1). A maximum of five colonies were picked up from each sample and subjected to further analysis. Genotyping by ERIC (enterobacterial repetitive intergenic consensus sequence)-PCR to assess the intra-sample diversity of isolates found the presence of multiclonal *E. albertii* in 30 (76.9%) of these PCR-positive samples (Table 1). Two distinct ERIC patterns were detected in 22 samples (56.4%), while one, three, and four patterns were found in 9 (23.1%), 6 (15.4%), and 2 (5.1%) samples, respectively. In total, 79 *E. albertii* strains were obtained from environmental water samples.

All PCR-positive specimens from which *E. albertii* could not be isolated were confirmed as positive by an additional species-specific PCR assay targeting a gene distinct from *Eacdt* (36) (data not shown), supporting that the initial PCR results were not false positive. This provides additional evidence that *E. albertii* was indeed present in these specimens.

### Detection of fecal bacteria in environmental water

To assess possible fecal contamination in environmental water, the presence of Enterobacterales was examined in samples collected after October 17, 2022 (54 samples, EW2212 to EW2348), using a culture-based method on Violet Red Bile Dextrose (VRBD) agar. Red colonies presumptively identified as Enterobacterales were detected in all 54 samples, with bacterial counts ranging from 0.7 to 217 CFU/mL (Table S1).

### Occurrence of *E. albertii* in wild raccoons

Fecal samples were collected from 122 wild raccoons in Osaka, Japan, between 2021 and 2023 (Table S2). *E. albertii* was detected in 68 samples (55.7%) using the *E. albertii*-specific real-time PCR assay (Table 1). *E. albertii* was isolated from 56 (82.4%) of the PCR-positive samples (Table 1). Genotyping of the isolates by ERIC-PCR identified two ERIC patterns in 12 samples, while a single ERIC pattern was identified in the remaining 44 samples, respectively. As a result, a total of 68 *E. albertii* strains were obtained from wild raccoon samples.

All PCR-positive raccoon specimens from which *E. albertii* could not be isolated were confirmed as positive by the additional species-specific PCR assay, supporting the presence of *E. albertii* in the specimens.

### Virulence gene profiling and EAO genotyping

*E. albertii* strains isolated from environmental water (*n* = 79) and raccoon (*n* = 68) samples were subjected to genetic typing of *E. albertii* O-antigens (EAO genotyping) and virulence gene analysis. The *E. albertii* strains exhibited diverse characteristics, which are described below.

PCR-based EAO genotyping revealed that the 79 environmental water strains were classified into 20 EAO genotypes (Table S3). These included seven strains of EAOg18; five each of EAOg5, EAOg16, and EAOg25; four each of EAOg11 and EAOg39; two each of EAOg19, EAOg31, and EAOg40; and one each of EAOg7, EAOg8, EAOg10, EAOg17, EAOg20, EAOg22, EAOg28, EAOg29, EAOg32, EAOg33, and EAOg35. However, 32 strains were untypable (EAOgUT) as no amplification was observed other than the internal controls in the PCR assays.

Similarly, the 68 raccoon strains were classified into 20 EAOg, including seven each of EAOg5, EAOg21, and EAOg38; five of each EAOg10 and EAOg25; three each of EAOg19 and EAOg34; two each of EAOg7, EAOg9, EAOg16, EAOg18, and EAOg29; and one each of EAOg1, EAOg2, EAOg8, EAOg20, EAOg26, EAOg30, EAOg36, and EAOg39 (Table S4). Thirteen strains were determined to be EAOgUT.

Virulence gene analysis revealed that all the strains from environmental water and raccoons were positive for *paa* and *eae* genes, in addition to *Eacdt*, which was used as marker for the detection and identification of *E. albertii* (Tables S4 and S5). Furthermore, *Eccdt-I* genes were detected in 19 strains from environmental water and 6 strains from raccoons. Among the *Eccdt-I* gene-positive strains, two strains from environmental water (EW2201-2 and EW2202-1) and one strain from a raccoon (RAC2244-1) were also positive for *stx2f* genes.

### Genotyping of *E. albertii* strains from sequentially collected water samples

Since ERIC-PCR generally has a lower discriminatory power for differentiating bacterial clones compared to classical typing methods, such as pulsed-field gel electrophoresis (PFGE), we primarily used this PCR assay to assess the intra-sample diversity of *E. albertii* isolates in this study. However, we also applied the ERIC-PCR assay to evaluate inter-sample diversity, restricting this comparison to strains obtained from sequential samples (Fig. S1). As expected, certain genotypes of *E. albertii* were consistently detected over time, along with the isolation of additional genotypes. An exception was observed in the samples collected on September 4, 2023, at location B (samples EW2344 to EW2346). In the first sample (EW2344), two genotypes (I and II) were identified. The second sample identified three genotypes (I, II, and III). However, in the third sample, only genotype III was detected, and genotypes I and II were not detected.

One strain representing each ERIC type from each sequential sample was selected and subjected to whole-genome sequencing analysis. For example, strains EW2330-1, EW2330-2, and EW2331-2 were selected from those isolated from samples on May 12, 2023 (Table S3).

### Whole-genome-based characteristics of *E. albertii* strains

A total of 119 selected *E. albertii* strains, including 61 from environmental water and 58 from raccoons, were subjected to whole-genome sequencing using the Illumina short-read platform. The assembled draft genome of all 119 strains showed >98% average nucleotide identity (ANI) with the reference genome of *E. albertii* strain CB9786, whereas the ANI with *E. coli* K-12 strain MG1655 was approximately 90% (Tables S3 and S4). These results further confirmed the identification of the strains as *E. albertii*.

*In silico* EAO genotyping based on whole-genome sequences (WGSs) was consistent with results obtained using PCR-based EAO genotyping. Virulence gene profiling, conducted using the Virulence Factor Database analyzer, also corresponded to the PCR detection of *eae*, *paa*, *Eccdt-I*, and *stx2f* genes (data not shown). Together, these WGS analyses confirmed that genetic profiling, including EAO genotypes and virulence gene identification, was accurately performed for all the 119 strains.

Next, the draft genome of the *E. albertii* strains was subjected to antimicrobial resistance (AMR) gene profiling using ResFinder with the *E. coli* database. No AMR gene was identified among the 119 strains. Consistently, 17 randomly selected *E. albertii* strains from environmental water (10 strains: EW2207-1, EW2209-1, EW2210-1, EW2213-2, EW2216-1, EW2305-1, EW2311-2, EW2335-1, EW2344-2, EW2347-1) and raccoons (7 strains: RAC2179-1, RAC2186-1, RAC2196-1, RAC2199-1, RAC2289-1, RAC2295-2, RAC2356-1) were found to be susceptible to 17 tested antimicrobials, which covered 9 classes: penicillins, cephalosporins, fosfomycin, carbapenems, aminoglycosides, amphenicols, tetracycline, quinolones, and sulfamethoxazole-trimethoprim, as listed in the “Materials and Methods” section (data not shown).

Plasmid replicon gene typing of the strains was also performed using PlasmidFinder (Tables S3 and S4). Twenty-three strains from each of the environmental water and raccoon sources did not carry any plasmid replicons. In contrast, 38 environmental water strains and 35 raccoon strains carried one to four replicons. Among the environmental water strains, 11 distinct plasmid replicons were identified. The most frequently detected replicon was IncFIB(AP001918), which was detected in 26 strains, followed by IncFII in 11 strains, pO111 in 7, IncFIC(FII) in 5, IncFII(pHN7A8) and IncI(Gamma) in 2 each, and Col(pHAD28), IncFIA(HI1), IncFII(pCoo), IncY, and IncI2(Delta) in 1 strain each. Fifteen distinct plasmid replicons were detected in the raccoon strains. IncFIB(AP001918), IncFII, and IncFIC(FII) were also frequently observed, which were identified in 31, 11, and 7 raccoon strains, respectively. IncFIB(AP001918) often co-existed with IncFII(pSE11), IncFIC(FII), and IncFII, including IncFII, IncFII(HI1), IncFII(pHN7A8), and IncFII (29), among both environmental water and raccoon *E. albertii* strains.

### Comparative genomic analysis among *E. albertii* from environmental water, raccoons, and diarrheal humans

**Fig 2:**
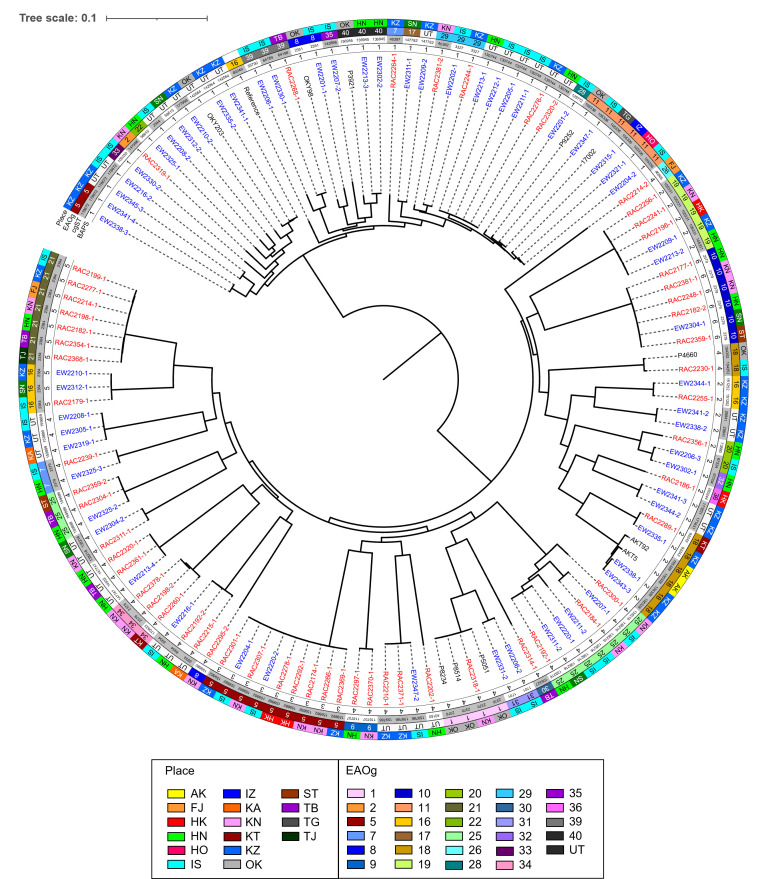
Genomic characteristics and phylogenetic relatedness of *Escherichia albertii* strains isolated from environmental water, wild raccoons, and patients in Japan. Maximum likelihood phylogenetic tree was constructed based on the 16,175 core genome SNPs of selected 130 *E. albertii* strains, including 61 environmental water, 58 raccoon, and 11 patient origins. The mid-point rooted tree is shown with strain names, sampling information, and genomic characteristics. Strain names of environmental water and raccoon origins were highlighted by blue and red, respectively. *E. albertii* strain CB9786 was used as a reference strain (accession number AP014856.1). cgST of the isolates was determined by cgMLST Finder v1.0.1 with *E. coli* database. O-antigen genotypes were determined by *in silico* analysis using whole-genome sequences of the strains. Bayesian analysis of population structure (BAPS) was performed using RhierBAPS v1.1.3. Abbreviations of places: AK, Akita; FJ, Fujiidera; HK, Hirakata; HN, Hannan; HO, Higashiosaka; IS, Izumisano; IZ, Izumi; KA, Kanan; KN, Kawachinagano; KT, Katano; KZ, Kaizuka; OK, Okayama; ST, Settsu; TB, Tondabayashi; TG, Tochigi; TJ, Tajiri.

The 119 *E. albertii* strains isolated from environmental water and raccoons were subjected to in-depth comparative genomic analysis to understand the diversity and phylogenetic relationships of the strains isolated in this study (Fig. 2).

First, the *E. albertii* strains were analyzed by core genome multi-locus sequence typing (cgMLST) using the *E. coli* database (Fig. 2; Tables S3 and S4). The 61 environmental water and 58 raccoon strains were classified into 32 and 29 diverse cgSTs, respectively, and even the strains belonging to the same EAOg showed the diversity of cgSTs. For example, two cgSTs were identified in the environmental water strains belonging to each EAOg5 (159882 and 178672), EAOg16 (2384 and 151252), EAOg18 (92242 and 155328), EAOg25 (138216 and 159889), and EAOg39 (55790 and 64189). Similarly, in the raccoon strains, two cgSTs were found in each EAOg7 (2379 and 40397), EAOg18 (92242 and 104352), EAOg29 (3327 and 40392), and EAOg34 (2378 and 170833).

**Fig 3:**
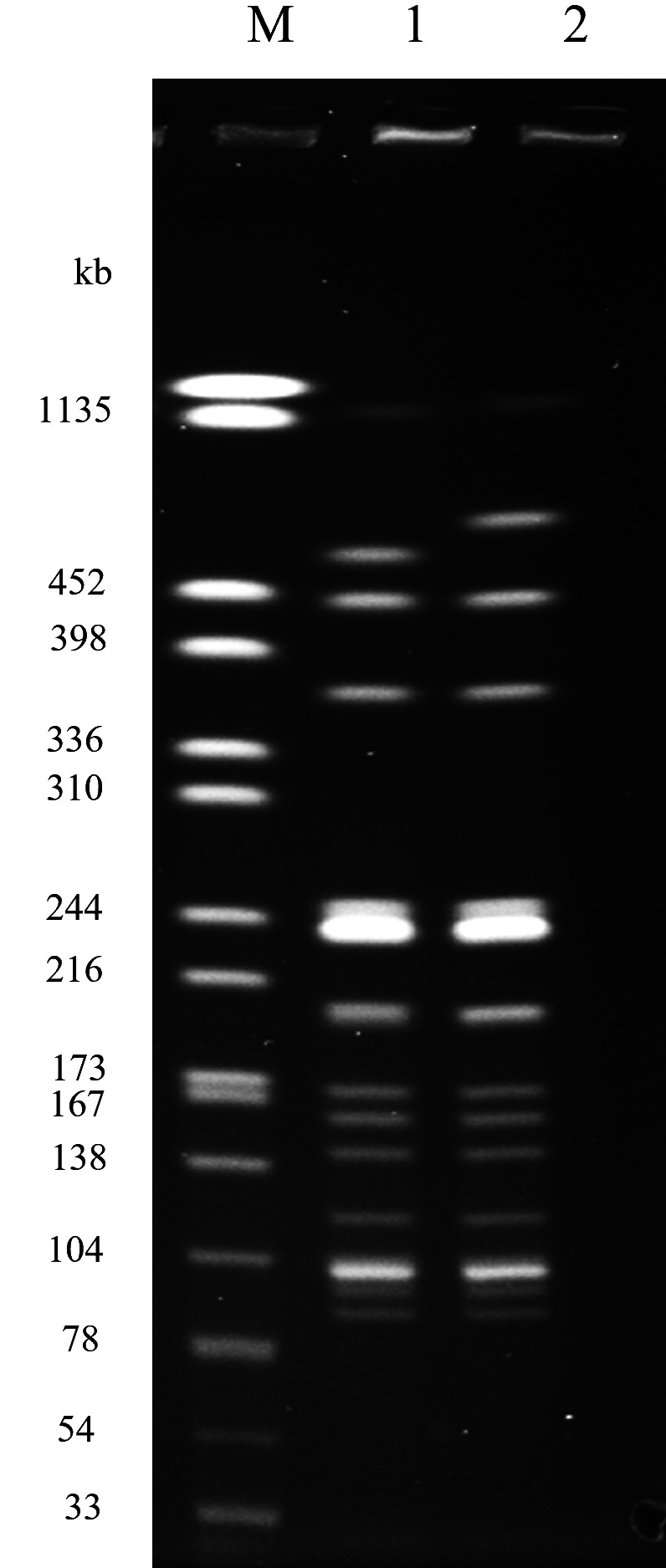
DNA fingerprints of *E. albertii* strains isolated from an environmental water and a raccoon. Genomic DNA was digested with *Xba*I and separated by pulsed-field gel electrophoresis. Lanes 1, EW2213-4 (environmental water); 2, RAC2320-1 (raccoon). M, *Xba*I-digested genomic DNA of *Salmonella* Braenderup strain H9812 used as a molecular size marker.

Next, we analyzed the phylogenetic relationship of *E. albertii* strains isolated from environmental water and raccoons, based on their core genome single-nucleotide polymorphisms (cgSNPs) (Fig. 2). The *E. albertii* strains grouped into two main clades, each containing several distinct lineages, indicating substantial phylogenetic diversity. Interestingly, strains from water and raccoons often overlapped, and many strains were either identical or closely related across and within the two sources (Fig. 2). Pairwise SNP distance analysis found several examples of strains with clonal relationships (Table S5), including 1 SNP (EW2202-1 and RAC2244-1), 4–14 SNPs (EW2205-1, EW2211-1, EW2212-1, EW2213-1, RAC2276-1, and RAC2320-2), 13 SNPs (EW2204-2 and EW2321-1), 1–5 SNPs (EW2209-1, EW2213-2, and RAC2196-1), 3–12 SNPs (EW2304-1, RAC2177-1, RAC2182-2, RAC2248-1, and RAC2381-1), 0–1 SNPs (EW2220-1, EW2311-2, and RAC2192-1), 0–1 SNPs (EW2347-2, RAC2210-1, and RAC2371-1), 1 SNP (EW2204-1 and RAC2301-1), 0 SNPs (EW2216-1 and RAC2192-2), 1 SNP (RAC2198-2 and RAC2378-1), 3–4 SNPs (EW2213-4, RAC2320-1, and RAC2361-1), 3 SNPs (EW2325-3 and RAC2239-1), 0–2 SNPs (EW2208-1, EW2305-1, and EW2319-1), 4–13 SNPs (EW2210-1, EW2312-1, and RAC2179-1), 6 SNPs (EW2330-1 and RAC2268-1), 9 SNPs (RAC2182-1 and RAC2368-1), and 4 SNPs (RAC2199-1 and RAC2277-1). Most of these clonally related strains exhibited identical plasmid profiles (Table S6). The clonal relationships were mainly observed among strains isolated from locations beyond city borders. Notably, one exception was strain pair EW2213-4 and RAC2320-1 (3 SNPs), isolated from river water and a raccoon, respectively, collected around the same time (October 2022) in the same city. These strains exhibited only a one-band difference in PFGE analysis (Fig. 3). According to Tenover’s criteria (37), they are considered clonally related.

In contrast, some clonally related strains exhibited differences in their plasmid profiles (Table S6). For example, strains EW2213-3 and EW2302, isolated from the same location 3 months apart, showed no SNP difference but had different plasmid profiles.

Although 36 closely related *E. albertii* clone pairs were observed between environmental water and raccoon samples, a permutation test (*n* = 1,000) indicated that this number did not exceed that expected by chance (*P* = 0.847), suggesting no significant enrichment of closely related clones between the two sources.

Consistent with ERIC-PCR–based typing, strains obtained from sequential samples showed either 0 or 2 SNP differences across their sampling dates, for example, EW2338-3 vs EW2341-4 (0 SNPs), EW2338-2 vs EW2341-2 (0 SNPs), EW2338-1 vs EW2343-3 (0 SNPs), and EW2335-2 vs EW2341-2 (2 SNPs) (Table S6). Each of these strain pairs also exhibited identical plasmid profiles.

In this analysis, 11 *E. albertii* strains clinically isolated from humans in Japan were included (Table S7). Some of these clinical strains clustered with strains isolated from environmental water and/or raccoon samples (Fig. 2). Pairwise SNP distance analysis revealed 23 SNP differences between water strain EW2347-1 and clinical strain 17,002 (Table S6). Furthermore, only 6 to 9 SNP differences were observed between the raccoon strain RAC2318-1 and the clinical strains P5051, P8234, and P8514.

## DISCUSSION

Although *E. albertii* is increasingly recognized as a zoonotic enteropathogen, its ecological reservoirs, environmental persistence, and transmission dynamics remain poorly understood. In this study, we performed integrated surveillance of environmental water and wild raccoons conducted contemporaneously within the same geographic area in Osaka, Japan, to address these knowledge gaps. Our study found a high prevalence of *E. albertii* in both river water (76.6%) and raccoons (55.7%), suggesting widespread environmental presence and wildlife carriage. Whole-genome sequencing and its comparative analyses of the strains demonstrated substantial genomic diversity and phylogenetic overlaps between strains from both sources, suggesting genetic relatedness. These findings contribute to our understanding of the environmental ecology of *E. albertii* and underscore the importance of a One Health approach to monitor this pathogen across human, animal, and environmental interfaces.

The prevalence of *E. albertii* in environmental water observed in this study (76.6%) was considerably higher than that reported in previous studies in Japan, which showed detection rates ranging from 0% to 33.3% (32, 38, 39). Although our recent pilot study in Tennessee, USA, detected *E. albertii* in 100% of river water samples using an *E. albertii*-specific PCR (18), this result should be interpreted cautiously due to the small sample size (*n* = 5). Several factors may explain the variation in detection rates, including geographical locations, seasonal differences, and methodological approaches. Among these, we speculate that methodological differences, particularly sample volume, might play a critical role in detection outcomes, as evidenced by the following observations. The detection rate of *E. albertii* by PCR from water samples was closely linked to the volume of water tested. Reported PCR-positive rates were 0%, 3.5%, 33.3%, and 100% when using 3 mL, 50–60 mL, 500 mL, and 1,000 mL, respectively (18, 32, 38, 39). This trend is consistent with quantitative estimates of *E. albertii* abundance in river water, which ranged from <5 to 71.7 cells per 100 mL based on the most probable number (MPN) method (39). Based on these findings, we selected a 1,000 mL sample volume in this study, which likely contributed to the higher observed prevalence of *E. albertii*. Taken together, these findings suggest that the true prevalence of *E. albertii* in environmental water may have been underestimated in earlier studies and highlight the importance of using adequate sample volumes to ensure reliable detection.

Understanding how environmental water becomes contaminated with *E. albertii* is essential for elucidating the ecology and transmission dynamics of this bacterium. Our extensive survey of environmental water revealed a widespread presence of *E. albertii* in riverine systems, including upper reaches and areas near river sources (Fig. 1). These regions are primarily inhabited by wild animals and are minimally affected by human activity, as they are located within non-human habitat ranges. Surface waters are known to be contaminated with fecal matter from wild animals (40, 41), and indeed, fecal bacteria were detected in all of the environmental water samples by culture with VBRD agar (Table S1). *E. albertii* has also been isolated from several wild animal species (2, 13, 17–19, 21, 23, 42). Furthermore, Xu et al. reported seasonal variation in *E. albertii* carriage among wild raccoons with a reduction in prevalence during the winter season in Japan (43). Consistent with this observation, the *E. albertii*-negative water samples in this study were primarily collected in winter. Taken together, these findings suggest that wild animals might play an important role in contaminating environmental water with this pathogen.

To investigate the association between *E. albertii* in wild animals and environmental water, we conducted raccoon surveillance in the same prefecture during a sampling period that overlapped with water sampling. Raccoons are highly adaptable omnivorous mammals native to North and Central America, and their populations and geographic ranges have expanded in many countries, including Japan, following their introduction for pet and commercial purposes (44–46) (https://www.env.go.jp/press/105902.html). Raccoons typically have a home range of several square kilometers, with reported ranges of 35 to 2,219 ha depending on habitat and food availability (47). They also frequently utilize aquatic environments for foraging and movement, as described in the Encyclopedia Britannica (https://www.britannica.com/animal/raccoon). In addition, raccoons share habitats with various wild animals (48), creating opportunities for indirect pathogen exchange through shared environmental resources such as surface water. The high prevalence of *E. albertii* detected in raccoons was consistent with previous studies conducted in Osaka, Japan, which reported 57.7% positivity among samples collected between 2016 and 2017, and 62% positivity between 2017 and 2020 (17, 43). Consistent with earlier studies (17), genetically diverse *E. albertii* strains were isolated from raccoons. Notably, the raccoon-derived *E. albertii* strains exhibited overlapping phylogenetic relationships and clonal similarities with strains isolated from environmental water. Particularly, clonally related *E. albertii* strains were identified in both water and a raccoon, sampled around the same time in the same city (strains EW2213-4 and RAC2320-1). Applying a 17-20 SNP cutoff that is commonly used to evaluate transmission and endogenous infections of *E. coli*, a close relative of *E. albertii* (49, 50), these findings indicate an epidemiological link between river water and raccoons. However, permutation testing showed that the frequency of such water–raccoon clone pairs was not higher than expected by chance. This indicates that co-occurrence alone does not provide statistical support for a directional source–sink relationship between raccoons and environmental water. Importantly, this study has two major limitations related to sampling design and methodology. First, raccoon samples were obtained from animals captured by multiple local municipalities as part of routine wildlife management programs, and sampling locations were therefore not selected or controlled for this study. Because precise geographic coordinates of raccoon capture sites were not available, it could not be determined whether the environmental water sampling sites fell within the home ranges of the captured raccoons. As a result, some of the water samples analyzed in this study may not have been directly influenced by fecal contamination from the sampled raccoon individuals. Second, *E. albertii* strains isolated from both raccoons and environmental water exhibited high genomic diversity. In addition, the *E. albertii* clones identified from individual raccoon specimens were shown to differ depending on the isolation methodology, particularly the enrichment procedures (51). These observations highlight the methodological limitation of selecting only a small number of colonies from each sample, which may not fully capture the clonal diversity present within individual specimens. Under these conditions, detecting statistically significant directional associations between specific animal hosts and environmental sources is inherently challenging. Therefore, while raccoons may contribute to environmental contamination, our data do not directly support the interpretation that raccoons represent the predominant source of *E. albertii* in environmental waters. Taken together with previous reports of *E. albertii* carriage not only in raccoons but also in wild birds and other animal species (2, 13, 17–19, 21, 23, 42), these findings suggest the need for comprehensive epidemiological investigations that consider sampling locations as well as multiple potential animal and environmental sources.

Clonal relationships were also observed among strains isolated from locations beyond city borders. For example, strain pairs, such as EW2202-2 and EW2321-1; RAC2268-1 and EW2330-1; and RAC2196-1, EW2209-1, and EW2213-2, were recovered from geographically distant sites, likely outside the typical habitat ranges of raccoons (48, 52, 53). Given that continuous riverine systems connecting the cities included in this study are generally absent, and considering that *E. albertii* has been identified in several migratory bird species (2, 20–23), it is plausible that wild birds may contribute to the dissemination of certain *E. albertii* clones across different locations. Further studies are warranted to evaluate this possibility. Moreover, environmental water strains EW2205-1, EW2212-1, and EW2213-1, along with raccoon strains RAC2276-1 and RAC2320-2, exhibited fewer than 17 SNP differences and were isolated from three neighboring cities. However, among these, strains EW2205-1, EW2213-1, and RAC2320-2 showed differences in plasmid profiles: they carried Col(pHAD28), IncY, and IncFIC(FII), respectively, in addition to IncFIB(AP001918) replicon. These data suggest that the *E. albertii* clones likely shared a common ancestor that was introduced into different environments and subsequently evolved over time. Collectively, these findings provide important insights into the ecology and transmission pathways of *E. albertii*.

Recent WGS-based studies have reported the genomic characteristics and phylogeny of *E. albertii* to better understand its ecology and transmission dynamics (54, 55). However, these studies have notable limitations, as they primarily relied on WGS data from publicly available databases, comprising *E. albertii* strains from geographically diverse, temporally dispersed, and epidemiologically unrelated sources. Therefore, to the best of our knowledge, this is the first report demonstrating prefecture-wide clonal relationships of *E. albertii*, at least among wildlife and environmental sources.

All *E. albertii* strains isolated in this study harbored the virulence genes *eae* and *paa*, which are known to be present in clinical strains (3), suggesting their potential to cause disease in humans. Additionally, some strains carried *stx2f* genes. Stx2 is a major virulence factor of EHEC, responsible for causing severe diseases in humans. Indeed, Stx2-producing *E. albertii* has been isolated from patients with bloody diarrhea and hemolytic uremic syndrome (7, 56), highlighting the higher virulence potential of Stx2-producing strains. Comparative genomic analysis further demonstrated that clinical strains isolated in Japan clustered within the same clades as strains derived from environmental water and raccoons. Interestingly, three clinical strains showed a clonal relationship with a raccoon-derived strain, differing by only 6–9 SNPs, despite differences in the years of sample collection. Furthermore, another clinical strain 17002 co-clustered with an environmental water strain, showing 23 SNP differences. Strain 17002 was isolated from a human outbreak in Tochigi, Japan, in 2017, which involved 137 cases (57). Notably, the affected individuals had participated in outdoor activities and were exposed to *E. albertii* through the consumption of meals that included agricultural products such as salads (57). Although direct evidence of *E. albertii* transmission among humans, raccoons, and environmental water is currently lacking, a 100-SNP cutoff has recently been proposed for evaluating cross-source genomic relatedness in the One Health context (58). Collectively, these findings suggest that pathogenic *E. albertii* are maintained in wildlife and environmental sources and could serve as reservoirs of human infection.

In conclusion, this study demonstrates that *E. albertii* is widely present in environmental water and that raccoons may contribute to environmental contamination by this pathogen. The observed prevalence, genetic diversity, and phylogenetic overlap among wildlife, environmental, and human clinical strains suggest ecological versatility of *E. albertii* and its potential relevance to public health. However, other wild animal species and environmental water sources, such as lakes and ponds, were not evaluated in this study. Therefore, systemic surveillance under a One Health framework, integrating human, a broader range of diverse animals, and diverse environmental interfaces, is required to fully understand and mitigate the transmission risk of *E. albertii*.

## MATERIALS AND METHODS

### Sample collection

One liter of environmental water sample was collected in 1 L storage bottles (#430518, Corning Inc., Corning, NY) from rivers, including stream (*n* = 11), creek (*n* = 27), brook (*n* = 22), and farm ditch (*n* = 3), as well as one spring in Osaka Prefecture, Japan, between August 2022 and October 2023. The sampling date and location are shown in Table S1 and Fig. 1. Samples EW2330 to EW2332, E2335 to EW2337, EW2338 to EW2340, EW2341 to EW2343, and EW2344 to EW2346 were collected three times at 30-min intervals at each location. Water flow velocity and displacement distances were not measured. Samples were transported to the laboratory of Osaka Metropolitan University at ambient temperature. However, the samples were kept on ice during transportation when they were collected during the summer season (>30°C air temperature) or they were unable to be placed in a refrigerator within 2 h of collection. The samples were kept at 4°C in a refrigerator and processed within 24 h of sampling.

As for raccoon fecal samples, rectal specimens were collected using cotton swabs (SEEDSWAB γ1; Eiken Chemical Co., Ltd., Tokyo, Japan) from 182 apparently healthy wild raccoon individuals (*Procyon lotor*) in Osaka, Japan, during November 2021 to March 2023. The sampling date and location are shown in Table S2. The raccoons were captured by municipal officials as part of extermination programs implemented by the municipal authorities under the national framework for controlling invasive alien species in Japan. Fecal sampling in the present study was approved by Osaka Prefectural Government and performed according to the Guidelines for Animal Experimentation of Osaka Prefectural Animal Protection and Livestock Division. The samples were kept at 4°C in a refrigerator and processed within 2 days of sampling.

### Detection, isolation, and identification of *E. albertii*

For water samples, 1 L of water was passed through a paper filter (ADVANTEC Co., Ltd., Tokyo, Japan) to remove debris and then filtered with 0.45 μm pore size MCE membrane filter (Merck Millipore, Burlington, MA) using a suction filtration system (Merck Millipore) to capture microbes on the membrane. The paper and membrane filters were aseptically cut into half and placed in 14 mL of trypticase soy broth (TSB; Becton, Dickinson and Company, Franklin Lakes, NJ) in 50-mL tube (AS ONE Corp., Osaka, Japan), respectively. After incubation at 37°C for 2 h with vigorous shaking, two different combinations of selective supplements were added to the cultures for subsequent selective enrichment as follows: the cultures from one half of paper and membrane filters were adjusted to the composition of cefixime-tellurite-deoxycholate–supplemented TSB (CTD-TSB; 0.05 mg/L cefixime, 2.5 mg/L potassium tellurite [C-T supplement; Thermo Fisher Scientific, Waltham, MA] [59], and 1.04 g/L sodium deoxycholate [Nacalai Tesque, Inc., Kyoto, Japan]). The other cultures were adjusted to modified composition of novobiocin-cefixime-tellurite–supplemented modified TSB (modified NCT-mTSB; 20 mg/L novobiocin [Kanto Chemical Col., Inc., Tokyo, Japan], 0.05 mg/L cefixime, 2.5 mg/L potassium tellurite [C-T supplement], 1.5 g/L bile salt No. 3 [Becton, Dickinson and Company]) (60). The supplemented cultures were incubated at 37°C for 16 h with vigorous shaking.

Raccoon rectal specimens were processed as described previously (59). Briefly, rectal swabs were suspended in 1 mL of phosphate-buffered saline (PBS, pH 7.0). Three hundred microliters of the suspension were inoculated into 3 mL of TSB and CTD-TSB and incubated at 37°C for 16 h with vigorous shaking.

DNA template was prepared from 100 µL of each enrichment culture and 200 µL of the raccoon sample suspension by an alkaline heat extraction method, respectively, as previously described (59). The DNA templates were subjected to the *Eacdt* gene-specific real-time PCR assay for the detection of *E. albertii* (61).

When a sample tested PCR-positive, a serial dilution of the corresponding original raccoon sample suspension was spread, and the enrichment culture was streaked on an *E. albertii*-differential medium, XRM-MacConkey agar, respectively (62). The agar plates were incubated at 37°C overnight (for 18–20 h). A maximum of five colorless colonies per plate were selected as suspect *E. albertii* isolates and streaked on MacConkey agar plate (Eiken Chemical Co., Ltd.) for single-colony isolation. The single colonies were examined by a conventional PCR assay targeting *Eacdt* genes (63), and the PCR-positive colonies were confirmed as *E. albertii*.

The *E. albertii* colonies were grown in 3 mL of Luria-Bertani broth (Nacalai Tesque, Inc.) at 37°C overnight (for 14–16 h), and the culture was stored with 25% glycerol at −80°C. DNA template was prepared from the cultures by the alkaline heat extraction method, and the template was subjected to a genotyping PCR assay targeting enterobacterial repetitive intergenic consensus sequence (ERIC-PCR) (64) to examine the intra-sample diversity of *E. albertii* isolates. One representative strain was selected from each ERIC type within each sample and subjected to subsequent analyses.

When *E. albertii* could not be isolated from PCR-positive specimens, DNA templates prepared from the specimens and their enrichment cultures were subjected to an additional *E. albertii*-specific PCR assay targeting a gene distinct from *Eacdt* (36).

### Enumeration of fecal bacteria in environmental water

Fifty milliliters of each environmental water sample were centrifuged at 3,800 × *g* for 15 min. After removing 49 mL of the supernatant, the pellet was resuspended in the residual 1 mL of supernatant, resulting in a 50-fold concentrated suspension. An aliquot of 100 µL of the concentrated suspension was spread on triplicate VRBD agar plates (Merck Millipore, Burlington, MA). The plates were incubated at 37°C for 24 h. Red colonies were counted as Enterobacterales, and the bacterial count in the original environmental samples was calculated based on the dilution factor.

### EAO genotyping, virulence gene profiling, and PFGE typing

EAO genotyping (EAOg1 to EAOg40) was performed by multiplex PCR assays developed by Ooka et al. (65). Virulence gene profiling was done by PCR assays targeting *eae*, *paa*, *stx1*, *stx2*, *stx2f*, and *Eccdt-I* genes, respectively (21).

Antimicrobial susceptibility of selected *E. albertii* strains was performed by a disk diffusion method using 17 antimicrobials, including ampicillin (10 μg) (penicillins), cephalothin (30 μg), cefuroxime (30 μg), cefotaxime (30 μg), ceftazidime (30 μg), and cefoxitin (30 μg) (cephalosporins); meropenem (10 μg) and imipenem (10 μg) (carbapenems); fosfomycin (50 μg), streptomycin (10 μg), kanamycin (30 μg), and gentamicin (10 μg) (aminoglycosides); tetracycline (30 μg) (tetracyclines); chloramphenicol (30 μg) (amphenicols); nalidixic acid (30 μg) and ciprofloxacin (5 μg) (quinolones); and sulfamethoxazole-trimethoprim (23.75 μg/1.25 μg), according to the Clinical and Laboratory Standards Institute (CLSI) guidelines (M02-A12). The sizes of the growth inhibition zones were interpreted based on the criteria of the CLSI M100-S29 for the family Enterobacterales. The disks were purchased from Becton, Dickinson and Company.

### Genomic analysis

Macrorestriction fragment patterns of genomic DNA of *E. albertii* strains were examined by PFGE using *Xba*I enzyme following the Centers for Disease Control and Prevention (CDC) PulseNet standard protocol for *E. coli. Salmonella* Braenderup strain H9812 was used as the universal size standard.

WGS of selected *E. albertii* strains was determined using Illumina short-read sequencer NextSeq1000 (Illumina, San Diego, CA). Genomic DNA was isolated from 1 mL fresh culture in LB broth by using DNeasy Blood & Tissue kit (QIAGEN, Venlo, the Netherlands) according to the manufacturer’s protocol for Gram-negative bacteria. Genomic DNA library was prepared with dual indexing using the QIAseq FX DNA Library kit (QIAGEN). The DNA library was subjected to a 300 bp paired-end sequence using the NextSeq 1000/2000 P1 600-cycle reagent cartridge (Illumina).

Fastp v0.24.0 was used for trimming and filtering of raw read sequences (66), and SKESA v2.5.1 was used for *de novo* assembling with the option of ‘--min_contig 250’ (67). The assembled sequences were applied to the following analyses as the draft genomes. ANI calculation was done through fastANI v1.34 using the genome sequences of *E. albertii* strain CB9786 (accession number AP014856.1) and *E. coli* strain K-12 strain MG1655 (U00096) as references (68). AMR gene and plasmid profiling was performed on the online platform of the Center for Genomic Epidemiology (https://www.genomicepidemiology.org/services/) using the ResFinder v4.7.2 (69, 70) and PlasmidFinder v2.1 with the Enterobacterales database (70, 71). EAO genotypes were determined by *in silico* analysis as described previously (26). Briefly, trimmed sequencing reads of each strain were mapped to *wzx*/*wzy* gene sequences of the 40 EAOgs (65) on CLC Genomics Workbench 24 (QIAGEN). For phylogenetic analysis, Snippy v3.1 was used to identify single-nucleotide polymorphisms (SNPs) in the genomes of the *E. albertii* strains and generate the SNP alignment using the genome of *E. albertii* strain CB9786 as reference. Prophage regions were identified using PHASTEST (https://phastest.ca/) and masked from the alignment. Recombinant regions were identified and removed using Gubbins v3.4 (72). The final core genome alignment of 16,175 bases was extracted with SNP-sites (73). A maximum likelihood phylogenetic tree was inferred under the best-fit model of TVM+G4 with 1,000 bootstrap replicates using RAxML Next Generation v1.2.2 (74). The mid-point rooted tree was visualized using iTOL ver. 6 (75) together with other characteristics and sample information of each strain. RhierBAPS v1.1.3 was used to identify the clusters of *E. albertii* strains using the alignment file (76, 77). To calculate SNP distance among the *E. albertii* strains, the strains were divided into six groups based on the identified clusters (BAPS 1 to 6), and respective core genome alignments were prepared as mentioned above. Pairwise SNP distance was calculated from the resulting core genome alignments (19,549 to 33,962 bp in length) using snp-dists (https://github.com/tseemann/snp-dists). Clinical *E. albertii* strains shown in Table S7 were included in the analysis for genomic comparisons.

### Statistical analysis

To assess whether closely related clones were non-randomly distributed among sources, a permutation test was performed using R. Strain pairs with SNP distances <20 were defined as closely related clones. Source labels were randomly permuted at the strain level (*n* = 1,000 permutations), while preserving the phylogenetic relationships and the number of isolates from each source. For each permutation, the number of closely related clone pairs observed between water and raccoon isolates was calculated and compared with the observed number.

## Data Availability

The sequencing data have been deposited in NCBI Sequence Read Archive under BioProject PRJDB35693.

## SUPPLEMENTAL MATERIAL

Supplemental material aem.00076-26-s0001.pdf Fig. S1; Tables S1 to S7.

ASM does not own the copyrights to Supplemental Material that may be linked to, or accessed through, an article. The authors have granted ASM a non-exclusive, world-wide license to publish the Supplemental Material files. Please contact the corresponding author directly for reuse.
