# Supplementary material for: Integrated study on the occurrence and genomic features of Escherichia albertii in environmental water and raccoons in Japan
Source: Appl Environ Microbiol. 2026 Mar 24;92(4):e00076-26. doi: 10.1128/aem.00076-26 (PMC13101514; doi:10.1128/aem.00076-26)
Supplement: Supplemental material — Fig. S1; Tables S1 to S7. [file aem.00076-26-s0001.pdf]

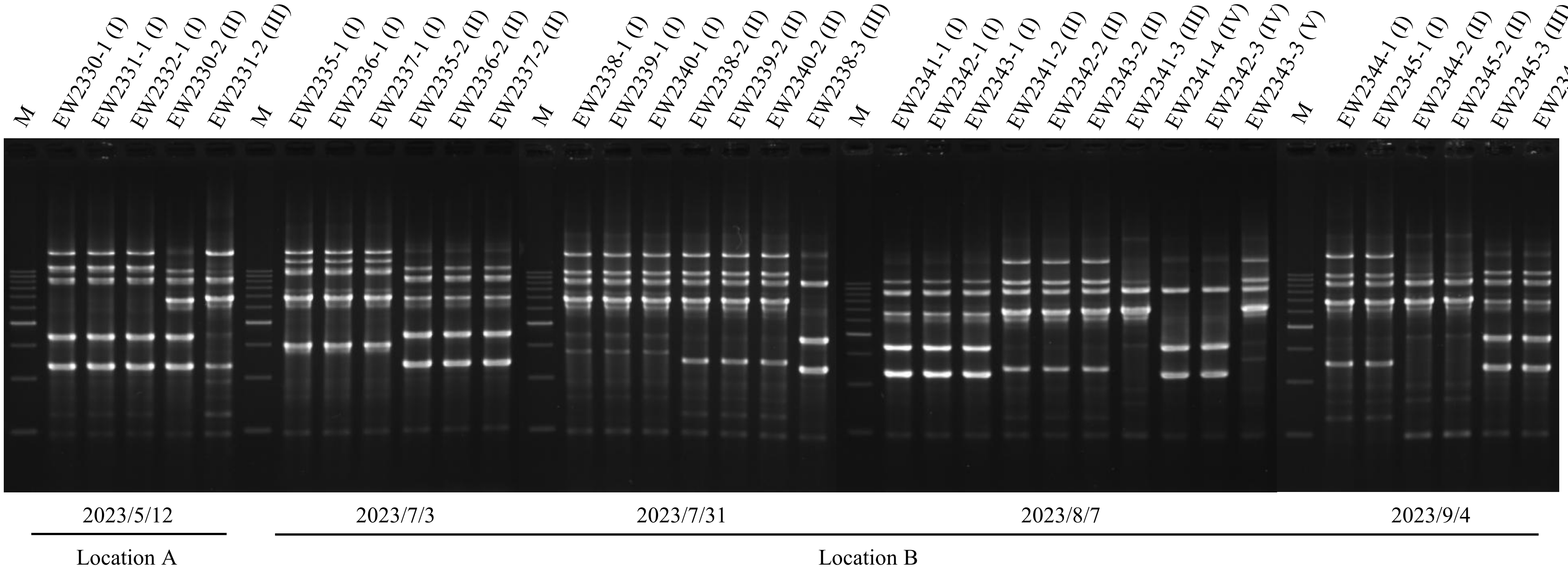

Fig S1 PCR-based typing of *Escherichia albertii* strains isolated from sequentially collected river water samples

River water was collected three times at intervals of 30 min at two locations (A and B) on each day: Samples EW2330 to EW2332 were collected at location A on 2023/5/12 (YYYY/MM/DD). Samples EW2335 to EW2337, EW2338 to EW2340, EW2341 to EW2343, and EW2344 to EW2346 were collected at location B on dates of 2023/7/3, 2023/7/31, 2023/8/7, and 2023/9/4, respectively. The *E. albertii* strains isolated from the water were subjected to PCR-based typing using the ERIC (enterobacterial repetitive intergenic consensus)-PCR assay. ERIC patterns obtained were defined for each sample (I to V) and shown in parenthesis with the strain ID. M indicates molecular weight marker of 500 bp DNA ladder.

Table S1 Detailed information of environmental river water samples analyzed in this study

No.	Sample ID	Location (latitude, longitude)	River	Riverine system	Fecal bacteria (CFU/100 mL)*	Detection	Isolation (No. of ERIC patterns)	Sampling date†
1	EW2201	34.37001, 135.32789	Stream	1	ND	+	yes (2)	2022/8/16
2	EW2202	34.37270, 135.33405	Stream	1	ND	+	yes (1)	
3	EW2203	34.37274, 135.34412	Stream	1	ND	+	no	2022/9/5
4	EW2204	34.36966, 135.34935	Stream	1	ND	+	yes (2)	
5	EW2205	34.35356, 135.37074	Creek	1	ND	+	yes (1)	2022/9/13
6	EW2206	34.3408, 135.37985	Brook	1	ND	+	yes (3)	
7	EW2207	34.33717, 135.39241	Brook	1	ND	+	yes (2)	2022/9/27
8	EW2208	34.34611, 135.40401	Brook	1	ND	+	yes (2)	
9	EW2209	34.36007, 135.42122	Creek	2	ND	+	yes (2)	2022/10/3
10	EW2210	34.34514, 135.41095	Brook	2	ND	+	yes (2)	
11	EW2211	34.32356, 135.37747	Creek	1	ND	+	yes (2)	
12	EW2212	34.37001, 135.32789	Stream	1	4800	+	yes (1)	2022/10/17
13	EW2213	34.3123, 135.27001	Creek	5	3900	+	yes (4)	
14	EW2214	34.37556, 135.34080	Farm ditch	5	467	+	no	2022/11/21
15	EW2215	34.37447, 135.33915	Farm ditch	5	2830	-	NA	
16	EW2216	34.36943, 135.33132	Farm ditch	1	420	+	yes (2)	2022/12/3
17	EW2217	34.38043, 135.29533	Stream	1	21700	+	no	
18	EW2218	34.36723, 135.43318	Creek	7	947	-	NA	2022/12/10
19	EW2219	34.35206, 135.43568	Brook	7	167	-	NA	
20	EW2220	34.36966, 135.34935	Stream	1	467	+	yes (2)	2022/12/17
21	EW2221	34.35356, 135.37074	Creek	1	2830	+	no	
22	EW2302	34.3123, 135.27001	Creek	3	120	+	yes (2)	2023/1/9
23	EW2303	34.30608, 135.26801	Creek	3	286	-	NA	
24	EW2304	34.33346, 135.32471	Creek	3	232	+	yes (2)	
25	EW2305	34.36208, 135.3979	Brook	2	2330	+	yes (1)	2023/1/17
26	EW2306	34.36243, 135.39839	Brook	2	834	-	NA	
27	EW2307	34.36488, 135.38971	Creek	2	3290	-	NA	
28	EW2308	34.33807, 135.38805	Brook	1	100	-	NA	2023/2/1
29	EW2309	34.33896, 135.38681	Spring	1	73	-	NA	
30	EW2310	34.33807, 135.38805	Brook	1	80	-	NA	
31	EW2311	34.35338, 135.31336	Creek	1	3180	+	yes (2)	2023/2/15
32	EW2312	34.35673, 135.31506	Creek	1	2860	+	yes (2)	
33	EW2313	34.38029, 135.45213	Brook	5	233	-	NA	2023/2/28
34	EW2314	34.38036, 135.45125	Creek	5	133	-	NA	
35	EW2315	34.39558, 135.45995	Creek	5	413	+	yes (1)	2023/3/21
36	EW2316	34.39505, 135.59399	Creek	4	2540	-	NA	
37	EW2317	34.43735, 135.62167	Creek	4	693	+	no	
38	EW2318	34.44636, 135.67172	Creek	4	120	-	NA	
39	EW2319	34.49639, 135.66448	Creek	4	6490	+	yes (1)	
40	EW2320	34.70655, 135.65747	Creek	6	10000	+	no	
41	EW2321	34.68354, 135.65432	Stream	6	1950	+	yes (1)	
42	EW2322	34.3123, 135.27001	Creek	3	520	-	NA	2023/4/3
43	EW2323	34.37001, 135.32789	Stream	1	286	+	no	
44	EW2324	34.2825, 135.13193	Creek	8	1190	-	NA	2023/4/24
45	EW2325	34.31328, 135.23117	Creek	3	1886	+	yes (3)	
46	EW2330	34.35356, 135.37074	Creek	1	633	+	yes (2)	2023/5/12
47	EW2331		Creek	1	613	+	yes (2)	
48	EW2332		Creek	1	1146	+	yes (1)	
49	EW2333	34.3123, 135.27001	Creek	3	3090	+	no	2023/6/15
50	EW2334	34.37001, 135.32789	Stream	1	3760	+	no	
51	EW2335	34.34514, 135.41095	Brook	2	186	+	yes (2)	2023/7/3
52	EW2336		Brook	2	274	+	yes (2)	
53	EW2337		Brook	2	346	+	yes (2)	
54	EW2338	34.34514, 135.41095	Brook	2	1200	+	yes (3)	2023/7/31
55	EW2339		Brook	2	894	+	yes (2)	
56	EW2340		Brook	2	400	+	yes (2)	
57	EW2341	34.34514, 135.41095	Brook	2	726	+	yes (4)	2023/8/7
58	EW2342		Brook	2	854	+	yes (3)	
59	EW2343		Brook	2	646	+	yes (3)	
60	EW2344	34.34514, 135.41095	Brook	2	220	+	yes (2)	2023/9/4
61	EW2345		Brook	2	374	+	yes (3)	
62	EW2346		Brook	2	1540	+	yes (1)	
63	EW2347	34.3536, 135.37076	Creek	1	2170	+	yes (2)	2023/10/7
64	EW2348	34.37017, 135.32389	Stream	1	2220	+	no	

†YYYY/MM/DD

*Fecal bacteria in environmental water samples were detected as red colonies on Violet Red Bile Dextrose (VRBD) agar.

Table S2 Detailed information of raccoon samples analyzed in this study

No.	Sample ID	Place where raccoon was captured	Detection	Isolation (No. of ERIC patterns)	Sampling date*
1	RAC2173	Hirakata city	+	yes (1)	2021/11/16
2	RAC2174	Kawachinagano city	+	yes (2)	
3	RAC2175	Hannan city	+	no	
4	RAC2176	Izumisano city	+	no	2021/11/30
5	RAC2177	Hannan city	+	yes (1)	
6	RAC2178	Tajiri town	+	no	
7	RAC2179	Izumisano city	+	yes (1)	2021/12/6
8	RAC2180	Hannan city	+	no	
9	RAC2181	Hannan city	+	no	
10	RAC2182	Hannan city	+	yes (2)	
11	RAC2183	Hirakata city	+	no	2021/12/14
12	RAC2184	Kawachinagano city	+	yes (1)	
13	RAC2185	Hannan city	+	no	
14	RAC2186	Hirakata city	+	yes (1)	2021/12/21
15	RAC2188	Kawachinagano city	-	NA	2022/1/25
16	RAC2189	Tondabayashi city	+	no	
17	RAC2190	Hannan city	+	no	
18	RAC2191	Hirakata city	-	NA	2022/2/1
19	RAC2192	Hannan city	+	yes (2)	
20	RAC2193	Izumiotsu city	-	NA	
21	RAC2194	Izumisano city	-	NA	2022/2/15
22	RAC2195	Minoh city	+	no	
23	RAC2196	Hirakata city	+	yes (1)	2022/2/22
24	RAC2197	Kanan town	-	NA	
25	RAC2198	Kawachinagano city	+	yes (2)	
26	RAC2199	Izumisano city	+	yes (1)	2022/3/2
27	RAC2200	Minoh city	+	no	
28	RAC2201	Kawachinagano city	-	NA	
29	RAC2202	Hannan city	+	yes (1)	
30	RAC2203	Kaizuka city	-	NA	
31	RAC2204	Izumisano city	-	NA	2022/3/16
32	RAC2205	Katano city	+	no	2022/3/23
33	RAC2206	Izumisano city	+	no	
34	RAC2207	Toyonaka city	-	NA	
35	RAC2208	Kawachinagano city	-	NA	
36	RAC2209	Izumisano city	-	NA	2022/4/6
37	RAC2210	Kaizuka city	+	yes (1)	
38	RAC2211	Hirakata city	-	NA	2022/4/13
39	RAC2212	Kaizuka city	-	NA	
40	RAC2213	Kawachinagano city	+	no	2022/4/20
41	RAC2214	Fujiidera city	+	yes (2)	
42	RAC2215	Kanan town	+	yes (1)	
43	RAC2216	Kaizuka city	-	NA	
44	RAC2217	Izumisano city	-	NA	2022/4/27
45	RAC2218	Minoh city	-	NA	
46	RAC2219	Tondabayashi city	-	NA	
47	RAC2220	Kawachinagano city	+	no	2022/5/11
48	RAC2221	Kawachinagano city	-	NA	
49	RAC2222	Kawachinagano city	-	NA	

50	RAC2223	Tondabayashi city	+	no	
51	RAC2224	Tondabayashi city	-	NA	
52	RAC2225	Tondabayashi city	-	NA	
53	RAC2226	Hannan city	-	NA	
54	RAC2227	Kaizuka city	+	yes (1)	
55	RAC2228	Izumisano city	-	NA	2022/5/18
56	RAC2229	Izumisano city	-	NA	
57	RAC2230	Izumisano city	+	yes (1)	
58	RAC2231	Katano city	-	NA	2022/5/25
59	RAC2232	Kawachinagano city	-	NA	
60	RAC2233	Kawachinagano city	-	NA	
61	RAC2234	Kawachinagano city	-	NA	
62	RAC2235	Kawachinagano city	-	NA	
63	RAC2236	Kawachinagano city	-	NA	
64	RAC2237	Kawachinagano city	-	NA	
65	RAC2238	Katano city	-	NA	2022/6/1
66	RAC2239	Izumisano city	+	yes (1)	
67	RAC2240	Kawachinagano city	-	NA	
68	RAC2241	Kawachinagano city	+	yes	
69	RAC2242	Tondabayashi city	-	NA	
70	RAC2243	Tondabayashi city	+	no	
71	RAC2244	Kaizuka city	+	yes (1)	
72	RAC2245	Katano city	+	yes (1)	2022/6/8
73	RAC2246	Kawachinagano city	-	NA	
74	RAC2247	Kawachinagano city	-	NA	
75	RAC2248	Kawachinagano city	+	yes (1)	
76	RAC2249	Minoh city	-	NA	
77	RAC2250	Tondabayashi city	-	NA	
78	RAC2251	Katano city	-	NA	2022/6/13
79	RAC2252	Katano city	-	NA	
80	RAC2253	Kawachinagano city	+	no	
81	RAC2254	Kaizuka city	-	NA	
82	RAC2255	Kaizuka city	+	yes (1)	
83	RAC2256	Kaizuka city	+	yes (1)	
84	RAC2257	Toyonaka city	-	NA	2022/6/20
85	RAC2258	Kaizuka city	+	no	
86	RAC2259	Hannan city	+	no	
87	RAC2260	Katano city	+	yes (1)	2022/6/27
88	RAC2261	Kawachinagano city	-	NA	
89	RAC2262	Tondabayashi city	-	NA	
90	RAC2263	Hannan city	+	no	
91	RAC2264	Kaizuka city	+	yes (1)	
92	RAC2265	Hannan city	-	NA	2022/7/4
93	RAC2266	Kawachinagano city	+	yes (1)	2022/7/11
94	RAC2267	Kawachinagano city	-	NA	
95	RAC2268	Tondabayashi city	+	yes (1)	
96	RAC2269	Kaizuka city	+	no	2022/7/25
97	RAC2270	Hannan city	-	NA	
98	RAC2271	Hannan city	+	no	
99	RAC2272	Hannan city	+	no	2022/8/1
100	RAC2273	Kawachinagano city	+	no	
101	RAC2274	Kawachinagano city	-	NA	2022/8/8

102	RAC2275	Kaizuka city	+	no	
103	RAC2276	Kaizuka city	+	yes (1)	
104	RAC2277	Kaizuka city	+	yes (1)	
105	RAC2278	Hirakata city	+	yes (1)	2022/8/15
106	RAC2279	Fujiidera city	+	no	
107	RAC2280	Hirakata city	-	NA	2022/8/22
108	RAC2281	Kawachinagano city	+	no	
109	RAC2289	Katano city	+	yes (1)	2022/9/5
110	RAC2290	Katano city	+	no	
111	RAC2291	Hirakata city	+	no	
112	RAC2292	Hirakata city	+	yes (1)	
113	RAC2293	Hirakata city	-	NA	
114	RAC2294	Kawachinagano city	+	no	
115	RAC2295	Kawachinagano city	+	yes (2)	
116	RAC2296	Tondabayashi city	-	NA	
117	RAC2297	Hannan city	+	yes (1)	
118	RAC2298	Hannan city	+	no	
119	RAC2299	Izumisano city	+	no	2022/9/12
120	RAC2300	Kawachinagano city	+	yes (1)	
121	RAC2301	Kaizuka city	+	yes (2)	
122	RAC2302	Kaizuka city	+	no	
123	RAC2303	Izumisano city	+	no	2022/9/27
124	RAC2304	Tondabayashi city	+	yes (2)	
125	RAC2305	Hannan city	+	no	
126	RAC2306	Kawachinagano city	+	no	
127	RAC2307	Kawachinagano city	+	yes (1)	2022/10/5
128	RAC2308	Hannan city	+	no	
129	RAC2309	Hannan city	+	no	
130	RAC2310	Izumisano city	+	no	2022/10/13
131	RAC2311	Kawachinagano city	+	yes (1)	
132	RAC2312	Kawachinagano city	+	no	
133	RAC2313	Toyonaka city	+	no	
134	RAC2314	Tondabayashi city	+	yes (1)	
135	RAC2315	Hannan city	+	no	
136	RAC2316	Izumisano city	+	no	2022/10/19
137	RAC2317	Izumisano city	+	no	
138	RAC2318	Kawachinagano city	+	yes (1)	
139	RAC2319	Kawachinagano city	+	yes (1)	
140	RAC2320	Hannan city	+	yes (2)	
141	RAC2321	Kaizuka city	+	no	
142	RAC2348	Izumisano city	+	no	2022/11/16
143	RAC2349	Kawachinagano city	+	no	
144	RAC2350	Izumisano city	+	no	2022/11/24
145	RAC2351	Kawachinagano city	+	no	
146	RAC2352	Kawachinagano city	+	no	
147	RAC2353	Toyonaka city	-	NA	
148	RAC2354	Tondabayashi city	+	yes (1)	
149	RAC2355	Kaizuka city	+	no	
150	RAC2356	Hannan city	+	yes (2)	
151	RAC2357	Hannan city	+	no	
152	RAC2358	Hirakata city	+	no	2022/11/29
153	RAC2359	Settsu city	+	yes (2)	

154	RAC2360	Kawachinagano city	+	no	
155	RAC2361	Tondabayashi city	+	yes (1)	
156	RAC2362	Kaizuka city	+	no	
157	RAC2363	Kaizuka city	+	no	
158	RAC2364	Tajiri town	+	yes (1)	
159	RAC2365	Hirakata city	+	no	2022/12/6
160	RAC2366	Kawachinagano city	-	NA	
161	RAC2367	Tondabayashi city	+	no	
162	RAC2368	Tajiri town	+	yes (1)	
163	RAC2369	Kaizuka city	+	yes (1)	
164	RAC2370	Kawachinagano city	+	yes (1)	2022/12/14
165	RAC2371	Kaizuka city	+	yes (1)	
166	RAC2372	Hirakata city	+	no	2022/12/21
167	RAC2373	Hannan city	-	NA	
168	RAC2377	Hirakata city	-	NA	2023/1/18
169	RAC2378	Kawachinagano city	+	yes (1)	
170	RAC2379	Kawachinagano city	+	no	
171	RAC2380	Kawachinagano city	+	no	
172	RAC2381	Kawachinagano city	+	yes (2)	2023/2/1
173	RAC2382	Kawachinagano city	-	NA	
174	RAC2383	Tondabayashi city	+	no	
175	RAC2384	Kaizuka city	-	NA	
176	RAC2385	Kawachinagano city	+	no	2023/3/1
177	RAC2386	Kawachinagano city	-	NA	
178	RAC2387	Kawachinagano city	-	NA	
179	RAC2388	Kawachinagano city	+	no	
180	RAC2389	Osaka-Sayama city	+	yes (1)	
181	RAC2390	Kawachinagano city	+	no	2023/3/6
182	RAC2391	Kawachinagano city	-	NA	

*YYYY/MM/DD

Table S3 Characteristics of the *E. albertii* strains isolated from enviromental water

Sample ID	Sampling location (latitude, longitude)	Sampling date	Strain ID	ERIC	EAOg	rhier BAPS	cgST[a]	Virulence genes other than *Eacdt*	Plasmid typing[b]	ANI[c]	
										Ea	*Ec*
EW22-1	34.37001, 135.32789	2022/8/16	EW2201-1	I	8	1	2351	*Eapaa* , *eae* , *Eccdt-I*	IncFIB(AP001918), pO111	99.2	90.2
			EW2201-2	II	28	1	129772	*Eapaa* , *eae* , *Eccdt-I* , *stx2f*	IncFIB(AP001918), pO111	99.2	90.2
EW22-2	34.37270, 135.33405		EW2202-1	I	29	1	3327	*Eapaa* , *eae* , *Eccdt-I* , *stx2f*	IncFIB(AP001918), pO111	99.3	90.2
EW22-4	34.36966, 135.34935	2022/9/5	EW2204-1	I	5	3	159882	*Eapaa* , *eae*	-	98.3	90.0
			EW2204-2	II	11	1	105336	*Eapaa* , *eae* , *Eccdt-I*	IncFIB(AP001918)	99.2	90.2
EW22-5	34.35356, 135.37074	2022/9/13	EW2205-1	I	UT	1	130744	*Eapaa* , *eae* , *Eccdt-I*	IncFIB(AP001918), Col(pHAD28)	99.2	90.1
EW22-6	34.3408, 135.37985		EW2206-1	I	39	1	55790	*Eapaa* , *eae* , *Eccdt-I*	-	99.1	90.3
			EW2206-2	II	31	4	1701	*Eapaa* , *eae*	pO111	98.5	90.1
			EW2206-3	III	20	2	67334	*Eapaa* , *eae*	-	98.5	90.1
EW22-7	34.33717, 135.39241	2022/9/27	EW2207-1	I	25	4	138216	*Eapaa* , *eae*	-	98.3	90.1
			EW2207-2	II	35	1	143896	*Eapaa* , *eae*	IncFIB(AP001918), pO111, IncFIA(HI1)	99.2	90.2
EW22-8	34.34611, 135.40401		EW2208-1	I	UT	4	159889	*Eapaa* , *eae*	IncFIB(AP001918)	98.3	90.1
			EW2208-2	II	UT	1	2344	*Eapaa* , *eae*	-	99.2	90.3
EW22-9	34.36007, 135.42122	2022/10/3	EW2209-1	I	19	2	105331	*Eapaa* , *eae* , *bfpA*	IncFIB(AP001918), IncFII(pHN7A8)	98.3	90.2
			EW2209-2	II	UT	1	147782	*Eapaa* , *eae*	-	99.3	90.2
EW22-10	34.34514, 135.41095		EW2210-1	I	16	5	2384	*Eapaa* , *eae*	IncFIB(AP001918), IncFII	98.3	90.0
			EW2210-2	II	UT	1	177958	*Eapaa* , *eae*	IncFII(pCoo)	99.2	90.3
EW22-11	34.32356, 135.37747		EW2211-1	I	UT	1	130744	*Eapaa* , *eae* , *Eccdt-I*	IncFIB(AP001918)	99.2	90.2
			EW2211-2	II	25	4	138216	*Eapaa* , *eae*	IncFIB(AP001918)	98.4	90.1
EW22-12	34.37, 135.32801	2022/10/17	EW2212-1	I	UT	1	130744	*Eapaa* , *eae* , *Eccdt-I*	IncFIB(AP001918)	99.2	90.1
EW22-13	34.3123, 135.27001		EW2213-1	I	UT	1	130744	*Eapaa* , *eae* , *Eccdt-I*	IncFIB(AP001918), IncY	99.3	90.2
			EW2213-2	II	19	2	105331	*Eapaa* , *eae* , *bfpA*	IncFIB(AP001918), IncFII(pHN7A8)	98.3	90.1
			EW2213-3	III	40	1	136945	*Eapaa* , *eae* , *Eccdt-I*	IncFIC(FII), IncI2(Delta), pO111	99.3	90.2
			EW2213-4	IV	UT	4	147747	*Eapaa* , *eae*	IncFIB(AP001918), IncFIC(FII)	98.4	90.1
EW22-16	34.36943, 135.33132	2022/12/3	EW2216-1	I	UT	4	6934	*Eapaa* , *eae*	-	98.3	90.0
			EW2216-2	II	UT	1	178672	*Eapaa* , *eae*	IncFII	99.2	90.2
EW22-20	34.36957, 135.3493	2022/12/17	EW2220-1	I	25	4	138216	*Eapaa* , *eae* , *bfpA*	IncFIB(AP001918)	98.3	90.2
			EW2220-2	II	5	3	159882	*Eapaa* , *eae*	-	98.3	90.0
EW23-2	34.31219, 135.26979	2023/1/9	EW2302-1	I	32	2	67334	*Eapaa* , *eae*	-	98.5	90.1
			EW2302-2	II	40	1	136945	*Eapaa* , *eae* , *Eccdt-I*	IncFIC(FII), pO111	99.3	90.2
EW23-4	34.33346, 135.32471		EW2304-1	I	10	6	2379	*Eapaa* , *eae*	-	98.3	90.0
			EW2304-2	II	UT	4	138216	*Eapaa* , *eae*	IncFIB(AP001918), IncFIC(FII)	98.3	90.0
EW23-5	34.36208, 135.3979	2023/1/17	EW2305-1	I	UT	4	159889	*Eapaa* , *eae*	IncFIB(AP001918)	98.4	90.1
EW23-11	34.35338, 135.31336	2023/2/15	EW2311-1	I	17	1	147782	*Eapaa* , *eae*	-	99.3	90.3
			EW2311-2	II	25	4	138216	*Eapaa* , *eae* , *bfpA*	IncFIB(AP001918)	98.3	90.2
EW23-12	34.35673, 135.31506		EW2312-1	I	16	5	2384	*Eapaa* , *eae*	IncFIB(AP001918), IncFIC(FII)	98.4	90.0

			EW2312-2	II	UT	1	58615	*Eapaa* , *eae*	-	99.2	90.2
EW23-15	34.39558, 135.45995	2023/3/21	EW2315-1	I	11	1	105336	*Eapaa* , *eae* , *Eccdt-I*	IncFIB(AP001918)	99.3	90.2
EW23-19	34.49639, 135.66448		EW2319-1	I	UT	4	159889	*Eapaa* , *eae*	IncFIB(AP001918)	98.3	90.1
EW23-21	34.68354, 135.65432		EW2321-1	I	11	1	105336	*Eapaa* , *eae* , *Eccdt-I*	IncFIB(AP001918)	99.2	90.2
EW23-25	34.31328, 135.23117	2023/4/24	EW2325-1	I	22	1	58615	*Eapaa* , *eae*	-	99.2	90.3
			EW2325-2	II	25	4	159889	*Eapaa* , *eae*	-	98.3	90.1
			EW2325-3	III	7	4	2379	*Eapaa* , *eae*	IncFIB(AP001918), IncFII	98.2	90.0
EW23-30	34.35349, 135.37077	2023/5/12	EW2330-1	I	39	1	64189	*Eapaa* , *eae* , *Eccdt-I*	-	99.1	90.3
			EW2330-2	II	33	1	105330	*Eapaa* , *eae* , *Eccdt-I*	IncFIB(AP001918), IncFII	99.2	90.2
EW23-31			EW2331-1	I	39	NA	NA	NA	NA	NA	NA
			EW2331-2	III	31	4	1701	*Eapaa* , *eae*	-	98.5	90.0
EW23-32			EW2332-1	I	39	NA	NA	NA	NA	NA	NA
EW23-35	34.34514, 135.41095	2023/7/3	EW2335-1	I	18	2	92242	*Eapaa* , *eae*	IncFII	98.5	90.1
			EW2335-2	II	UT	1	142064	*Eapaa* , *eae*	IncFII	99.2	90.2
EW23-36			EW2336-1	I	18	NA	NA	NA	NA	NA	NA
			EW2336-2	II	UT	NA	NA	NA	NA	NA	NA
EW23-37			EW2337-1	I	18	NA	NA	NA	NA	NA	NA
			EW2337-2	II	UT	NA	NA	NA	NA	NA	NA
EW23-38	34.34514, 135.41095	2023/7/31	EW2338-1	I	18	2	155328	*Eapaa* , *eae*	IncFII	98.5	90.1
			EW2338-2	II	UT	2	28841	*Eapaa* , *eae*	-	98.5	90.0
			EW2338-3	III	5	1	178672	*Eapaa* , *eae*	-	99.2	90.3
EW23-39			EW2339-1	I	18	NA	NA	NA	NA	NA	NA
			EW2339-2	II	UT	NA	NA	NA	NA	NA	NA
EW23-40			EW2340-1	I	18	NA	NA	NA	NA	NA	NA
			EW2340-2	II	UT	NA	NA	NA	NA	NA	NA
EW23-41	34.34514, 135.41095	2023/8/7	EW2341-1	I	UT	1	142064	*Eapaa* , *eae*	IncFII	99.3	90.2
			EW2341-2	II	UT	2	28841	*Eapaa* , *eae*	-	98.5	90.1
			EW2341-3	III	UT	2	171831	*Eapaa* , *eae*	-	98.3	90.1
			EW2341-4	IV	5	1	178672	*Eapaa* , *eae*	-	99.3	90.3
EW23-42			EW2342-1	I	UT	NA	NA	NA	NA	NA	NA
			EW2342-2	II	UT	NA	NA	NA	NA	NA	NA
			EW2342-3	IV	5	NA	NA	NA	NA	NA	NA
EW23-43			EW2343-1	I	UT	NA	NA	NA	NA	NA	NA
			EW2343-2	II	UT	NA	NA	NA	NA	NA	NA
			EW2343-3	V	18	2	155328	*Eapaa* , *eae*	IncFII	98.5	90.1
EW23-44	34.34514, 135.41095	2023/9/4	EW2344-1	I	16	2	151252	*Eapaa* , *eae*	-	98.4	90.2
			EW2344-2	II	UT	2	171831	*Eapaa* , *eae*	IncFIB(AP001918)	98.3	90.1
EW23-45			EW2345-1	I	16	NA	NA	NA	NA	NA	NA
			EW2345-2	II	UT	NA	NA	NA	NA	NA	NA

			EW2345-3	III	UT	1	178672	*Eapaa* , *eae* , *Eccdt-I*	IncFII, IncI(Gamma)	99.2	90.2
EW23-46			EW2346-1	I	16	NA	NA	NA	NA	NA	NA
EW23-47	34.3536, 135.37076	2023/10/7	EW2347-1	I	11	1	105336	*Eapaa* , *eae* , *Eccdt-I*	IncFII, IncI(Gamma)	99.3	90.3
			EW2347-2	II	UT	4	159786	*Eapaa* , *eae*	-	98.4	90.1

[a]cgST was identified from draft genome of each strain using cgMLSTFinder (v1.2) with *E. coli* database. -, no whole genome sequencing was done.

[b]Plasmid detection and typing was done using Plasmid Finder 2.1 with the Enterobacteriales database (https://cge.food.dtu.dk/services/PlasmidFinder/). '-' indicates no plasmid detected.

[c]Average nucleotide identitiy was calculated using FastANI software between WGS of the *E. albertii* isolates, *E. albertii* strain CB9786 (*Ea* ; accession number AP014856) and *E. coli* strain K-12 MG1655 (*Ec* ; U00096), respectively.

Table S4 Characteristics of the *E. albertii* strains isolated from raccoon samples

Sample ID	Sampling location	Sampling date	Strain ID	ERIC	EAOg	rhier BAPS	cgST[a]	Virulence genes other than *Eacdt*	Plasmid typing	ANI	
										Ea	*Ec*
RAC2173	Hirakata city	2021/11/16	RAC2173-1	I	38	NA	NA	*Eapaa* , *eae*	NA	NA	NA
RAC2174	Kawachinagano city		RAC2174-1	I	5	3	159882	*Eapaa* , *eae*	-	98.3	90.0
			RAC2174-2	II	UT	NA	NA	*Eapaa* , *eae*	NA	NA	NA
RAC2177	Hannan city	2021/11/30	RAC2177-1	I	10	6	2379	*Eapaa* , *eae*	ColpVC	98.3	90.0
RAC2179	Izumisano city	2021/12/7	RAC2179-1	I	16	5	2384	*Eapaa* , *eae*	IncFIB(AP001918), IncFII, IncI1-I(Alpha)	98.4	90.0
RAC2182	Hannan city		RAC2182-1	I	21	5	2384	*Eapaa* , *eae*	Col156	98.4	90.2
			RAC2182-2	II	10	6	2379	*Eapaa* , *eae*	-	98.3	90.0
RAC2184	Kawachinagano city	2021/12/14	RAC2184-1	I	25	4	138216	*Eapaa* , *eae*	-	98.3	90.1
RAC2186	Hirakata city	2021/12/16	RAC2186-1	I	36	2	203244	*Eapaa* , *eae*	IncFIB(AP001918), IncFII, IncFIA	98.4	90.0
RAC2192	Hannan city	2022/2/1	RAC2192-1	I	25	4	138216	*Eapaa* , *eae*	IncFIB(AP001918)	98.3	90.2
			RAC2192-2	II	UT	4	6934	*Eapaa* , *eae*	-	98.3	90.0
RAC2196	Hirakata city	2022/2/22	RAC2196-1	I	19	2	105331	*Eapaa* , *eae*	IncFIB(AP001918), IncFII(pHN7A8)	98.3	90.1
RAC2198	Kawachinagano city	2022/2/22	RAC2198-1	I	21	5	2384	*Eapaa* , *eae*	IncFIB(AP001918), IncFII	98.4	90.1
			RAC2198-2	II	34	4	2378	*Eapaa* , *eae*	IncFIB(AP001918), IncFIC(FII)	98.4	90.1
RAC2199	Izumisano city	2022/3/2	RAC2199-1	I	21	5	2384	*Eapaa* , *eae*	IncFIB(AP001918), IncFII, IncX1	98.4	90.1
RAC2202	Hannan city		RAC2202-1	I	UT	4	40155	*Eapaa* , *eae*	-	98.3	90.1
RAC2210	Kaizuka city	2022/4/6	RAC2210-1	I	UT	4	159786	*Eapaa* , *eae*	-	98.3	90.0
RAC2214	Fujiidera city	2022/4/20	RAC2214-1	I	21	5	2384	*Eapaa* , *eae*	IncFIB(AP001918), IncFII	98.4	90.1
			RAC2214-2	II	26	4	46865	*Eapaa* , *eae*	IncFIB(AP001918), IncFII	98.4	90.1
RAC2215	Kawaminami town		RAC2215-1	I	UT	4	6934	*Eapaa* , *eae*	-	98.3	90.0
RAC2227	Kaizuka city	2022/5/11	RAC2227-1	I	38	NA	NA	*Eapaa* , *eae*	NA	NA	NA
RAC2230	Izumisano city	2022/5/18	RAC2230-1	I	18	4	104352	*Eapaa* , *eae*	IncFIB(AP001918)	98.4	90.1
RAC2239	Izumisano city	2022/6/1	RAC2239-1	I	7	4	2379	*Eapaa* , *eae*	IncFIB(AP001918), IncFII	98.3	90.1
RAC2241	Kawachinagano city		RAC2241-1	I	19	2	105331	*Eapaa* , *eae*	IncFIB(AP001918), IncFII(pHN7A8)	98.3	90.1
RAC2244	Kaizuka city		RAC2244-1	I	29	1	3327	*Eapaa* , *eae* , *Eccdt-I* , *stx2f*	IncFIB(AP001918), pO111	99.3	90.2
RAC2245	Katano city	2022/6/8	RAC2245-1	I	38	NA	NA	*Eapaa* , *eae*	NA	NA	NA
RAC2248	Kawachinagano city		RAC2248-1	I	10	6	2379	*Eapaa* , *eae*	-	98.3	90.0
RAC2254	Kaizuka city	2022/6/14	RAC2254-1	I	38	NA	NA	*Eapaa* , *eae*	NA	NA	NA
RAC2255	Kaizuka city	2022/6/13	RAC2255-1	I	16	2	151252	*Eapaa* , *eae*	IncFII(pCoo), IncI(Gamma)	98.5	90.0
RAC2256	Kaizuka city		RAC2256-1	I	19	2	105331	*Eapaa* , *eae*	IncFIB(AP001918), IncFII(pHN7A8), Col156	98.3	90.1
RAC2260	Katano city	2022/6/27	RAC2260-1	I	34	4	170833	*Eapaa* , *eae*	-	98.4	90.1
RAC2264	Kaizuka city		RAC2264-1	I	7	1	40397	*Eapaa* , *eae* , *Eccdt-I*	IncFIB(AP001918), IncFII(29), IncFII(pHN7A8), pO111	99.2	90.3
RAC2266	Kawachinagano city	2022/7/11	RAC2266-1	I	5	3	159882	*Eapaa* , *eae*	-	98.4	90.0
RAC2268	Tondabayashi city		RAC2268-1	I	39	1	64189	*Eapaa* , *eae* , *Eccdt-I*	-	99.1	90.2
RAC2276	Kaizuka city	2022/8/8	RAC2276-1	I	UT	1	130744	*Eapaa* , *eae* , *Eccdt-I*	IncFIB(AP001918)	99.2	90.2
RAC2277	Kaizuka city		RAC2277-1	I	21	5	2384	*Eapaa* , *eae*	IncFIB(AP001918), IncFII	98.4	90.1
RAC2278	Hirakata city	2022/8/15	RAC2278-1	I	5	3	159882	*Eapaa* , *eae*	-	98.4	90.0
RAC2289	Katano city	2022/9/5	RAC2289-1	I	18	2	92242	*Eapaa* , *eae*	IncFIB(AP001918), IncFII(pHN7A8), Col156, ColpVC	98.5	90.1
RAC2292	Hirakata city		RAC2292-1	I	5	3	159882	*Eapaa* , *eae*	-	98.4	90.0
RAC2295	Kawachinagano city		RAC2295-1		UT	NA	NA	*Eapaa* , *eae*	NA	NA	NA
			RAC2295-2		8	4	159880	*Eapaa* , *eae*	IncFIB(AP001918), IncFIC(FII), Col156, ColpVC	98.3	90.1
RAC2297	Hannan city		RAC2297-1	I	9	4	118707	*Eapaa* , *eae*	-	98.4	90.0

RAC2300	Kawachinagano city	2022/9/12	RAC2300-1	I	25	4	138216	*Eapaa* , *eae*	IncFIB(AP001918)	98.3	90.1
RAC2301	Kaizuka city	2022/9/26	RAC2301-1	I	5	3	159882	*Eapaa* , *eae*	Col156, ColpVC, IncFII	98.4	90.0
			RAC2301-2	II	38	NA	NA	*Eapaa* , *eae*	NA	NA	NA
RAC2304	Tondabayashi city		RAC2304-1	I	25	4	159889	*Eapaa* , *eae*	-	98.4	90.1
RAC2307	Kawachinagano city	2022/10/6	RAC2307-1	I	5	3	159882	*Eapaa* , *eae*	-	98.4	90.0
RAC2311	Kawachinagano city	2022/10/11	RAC2311-1	I	UT	4	138216	*Eapaa* , *eae*	-	98.4	90.0
RAC2314	Tondabayashi city		RAC2314-1	I	30	4	105327	*Eapaa* , *eae* , *Eccdt-I*	IncFIB(AP001918), IncFII(pSE11), pO111	98.4	90.1
RAC2318	Kawachinagano city	2022/10/18	RAC2318-1	I	1	4	2370	*Eapaa* , *eae*	-	98.4	90.0
RAC2319	Kawachinagano city		RAC2319-1	I	2	1	147996	*Eapaa* , *eae*	-	99.1	90.3
RAC2320	Hannan city		RAC2320-1	I	UT	4	147747	*Eapaa* , *eae*	IncFIB(AP001918), IncFIC(FII)	98.4	90.1
			RAC2320-2	II	UT	1	130744	*Eapaa* , *eae* , *Eccdt-I*	IncFIB(AP001918)	99.2	90.2
RAC2354	Tondabayashi city	2022/11/24	RAC2354-1	I	21	5	2384	*Eapaa* , *eae*	IncFIB(AP001918), IncFII	98.4	90.1
RAC2356	Hannan city	2022/11/12	RAC2356-1	I	20	2	73880	*Eapaa* , *eae*	IncFIB(AP001918), IncFII(29)	98.4	90.1
			RAC2356-2	II	UT	NA	NA	*Eapaa* , *eae*	NA	NA	NA
RAC2359	Settsu city	2022/11/29	RAC2359-1	I	10	6	2379	*Eapaa* , *eae*	-	98.3	90.0
			RAC2359-2	II	25	4	159889	*Eapaa* , *eae*	-	98.4	90.1
RAC2361	Tondabayashi city		RAC2361-1	I	UT	4	14932	*Eapaa* , *eae*	IncFIB(AP001918), IncFIC(FII)	98.4	90.1
RAC2364	Tajiri town		RAC2364-1	I	38	NA	NA	*Eapaa* , *eae*	NA	NA	NA
RAC2368	Tajiri town	2022/12/6	RAC2368-1	I	21	5	2384	*Eapaa* , *eae*	IncFIB(AP001918), IncFII	98.4	90.1
RAC2369	Kaizuka city		RAC2369-1	I	5	3	159882	*Eapaa* , *eae*	IncFIB(AP001918)	98.4	90.0
RAC2370	Kawachinagano city	2022/12/13	RAC2370-1	I	9	4	118707	*Eapaa* , *eae*	-	98.4	90.1
RAC2371	Kaizuka city		RAC2371-1	I	UT	4	159786	*Eapaa* , *eae*	IncFIB(AP001918), IncFIC(FII)	98.3	90.0
RAC2378	Kawachinagano city	2023/1/18	RAC2378-1	I	34	4	2378	*Eapaa* , *eae*	IncFIB(AP001918), IncFIB(H89-PhagePlasmid), IncFIC(FII)	98.3	90.1
RAC2381	Kawachinagano city	2023/1/31	RAC2381-1	I	10	6	2379	*Eapaa* , *eae*	-	98.3	90.0
			RAC2381-2	II	29	1	40392	*Eapaa* , *eae*	IncFIB(AP001918), IncFIC(FII)	99.3	90.2
RAC2389	Osaka-Sayama city	2023/3/1	RAC2389-1	I	38	NA	NA	*Eapaa* , *eae*	NA	NA	NA

[a]cgST was identified from draft genome of each strain using cgMLSTFinder (v1.2) with *E. coli* database. -, no whole genome sequencing was done.

[b]Plasmid detection and typing was done using Plasmid Finder 2.1 with the Enterobacteriales database (https://cge.food.dtu.dk/services/PlasmidFinder/). '-' indicates no plasmid detected.

[c]Average nucleotide identitiy was calculated using FastANI software between WGS of the *E. albertii* isolates, *E. albertii* strain CB9786 (*Ea* ; accession number AP014856) and *E. coli* strain K-12 MG1655 (*Ec* ; U00096), respectively.

Table S5-1 Pairwise cgSNP distance among *E. albertii* strains assigned to BAPS 1

	17002	EW2201-1	EW2201-2	EW2202-1	EW2204-2	EW2205-1	EW2206-1	EW2207-2	EW2208-2	EW2209-2	EW2210-2	EW2211-1	EW2212-1	EW2213-3	EW2213-1	EW2216-2	EW2302-2	EW2311-1	EW2312-2	EW2315-1	EW2321-1	EW2325-1	EW2330-1	EW2330-2	EW2335-2	EW2338-3	EW2341-1	EW2341-4	EW2345-3	EW2347-1	OKY203	OKY98	P3921	P9252	RAC2244-1	RAC2264-1	RAC2268-1	RAC2276-1	RAC2319-1	RAC2320-2	RAC2381-2	Reference
17002		3507	369	866	325	546	3805	3169	3088	1948	3125	544	544	2883	550	2844	2883	1939	3031	350	328	3122	3574	3224	3148	2888	3150	2888	2843	23	3190	3033	2862	206	865	2893	3574	545	3405	546	722	2986
EW2201-1	3507		3538	3573	3532	3501	4583	3927	3971	3447	4080	3499	3499	3250	3505	3662	3250	3438	3880	3559	3537	3966	4364	4004	4090	3757	4092	3757	3661	3508	4132	1481	3225	3537	3572	3889	4364	3500	4342	3501	3428	3644
EW2201-2	369	3538		899	394	575	3834	3196	3119	1979	3156	573	573	2914	579	2872	2914	1970	3060	419	397	3153	3603	3255	3179	2918	3181	2918	2872	370	3221	3060	2889	395	898	2923	3603	574	3432	575	751	3017
EW2202-1	866	3573	899		893	856	3881	3243	3180	2010	3213	854	854	2959	860	2927	2959	2001	3110	918	896	3203	3646	3307	3234	2961	3236	2961	2926	867	3276	3099	2934	894	1	2953	3646	855	3483	856	685	3054
EW2204-2	325	3532	394	893		569	3830	3194	3113	1975	3150	567	567	2910	573	2868	2910	1966	3058	135	13	3149	3601	3251	3175	2914	3177	2914	2867	326	3217	3060	2889	351	892	2920	3601	568	3430	569	749	3011
EW2205-1	546	3501	575	856	569		3801	3155	3078	1940	3115	8	4	2875	14	2834	2875	1931	3022	594	572	3113	3568	3216	3138	2878	3140	2878	2833	547	3180	3025	2854	572	855	2881	3568	9	3394	10	712	2978
EW2206-1	3805	4583	3834	3881	3830	3801		4286	3984	3813	3994	3799	3799	3964	3805	3851	3964	3804	3880	3855	3833	3982	1795	4043	4054	3896	4056	3896	3851	3806	4098	4118	3932	3830	3880	3957	1797	3800	4319	3801	3734	3968
EW2207-2	3169	3927	3196	3243	3194	3155	4286		3671	3078	3668	3153	3153	3248	3159	3408	3248	3069	3571	3219	3197	3638	4063	3621	3708	3506	3710	3506	3407	3170	3754	3465	3219	3195	3242	3571	4065	3154	3892	3155	3091	3421
EW2208-2	3088	3971	3119	3180	3113	3078	3984	3671		3032	2022	3076	3076	3257	3082	2543	3257	3023	1571	3140	3116	1805	3751	2526	2073	2423	2075	2423	2542	3089	2117	3498	3227	3115	3179	3622	3753	3077	2557	3078	3035	2778
EW2209-2	1948	3447	1979	2010	1975	1940	3813	3078	3032		3075	1938	1938	2789	1944	2831	2789	51	2964	2000	1978	3074	3584	3191	3136	2957	3138	2957	2830	1949	3178	2973	2764	1974	2009	2845	3586	1939	3340	1940	1863	2839
EW2210-2	3125	4080	3156	3213	3150	3115	3994	3668	2022	3075		3113	3113	3342	3119	2525	3342	3066	2050	3175	3153	2091	3770	2574	1222	2532	1224	2532	2524	3126	1266	3603	3320	3150	3212	3651	3772	3114	2625	3115	3066	2844
EW2211-1	544	3499	573	854	567	8	3799	3153	3076	1938	3113		6	2873	12	2832	2873	1929	3020	592	570	3111	3566	3214	3138	2876	3140	2876	2831	545	3180	3023	2852	570	853	2879	3566	1	3392	2	710	2976
EW2212-1	544	3499	573	854	567	4	3799	3153	3076	1938	3113	6		2873	12	2832	2873	1929	3020	592	570	3111	3566	3214	3138	2876	3140	2876	2831	545	3180	3023	2852	570	853	2879	3566	7	3392	8	710	2976
EW2213-3	2883	3250	2914	2959	2910	2875	3964	3248	3257	2789	3342	2873	2873		2879	2973	0	2780	3161	2935	2913	3264	3739	3278	3387	3079	3389	3079	2972	2884	3429	2773	967	2909	2958	3260	3739	2874	3631	2875	2814	2920
EW2213-1	550	3505	579	860	573	14	3805	3159	3082	1944	3119	12	12	2879		2838	2879	1935	3026	598	576	3117	3572	3220	3144	2882	3146	2882	2837	551	3186	3029	2858	576	859	2885	3572	13	3398	14	716	2982
EW2216-2	2844	3662	2872	2927	2868	2834	3851	3408	2543	2831	2525	2832	2832	2973	2838		2973	2822	2384	2893	2871	2543	3618	2540	2577	2052	2579	2052	31	2845	2621	3186	2942	2869	2926	3361	3620	2833	2897	2834	2779	2841
EW2302-2	2883	3250	2914	2959	2910	2875	3964	3248	3257	2789	3342	2873	2873	0	2879	2973		2780	3161	2935	2913	3264	3739	3278	3387	3079	3389	3079	2972	2884	3429	2773	967	2909	2958	3260	3739	2874	3631	2875	2814	2920
EW2311-1	1939	3438	1970	2001	1966	1931	3804	3069	3023	51	3066	1929	1929	2780	1935	2822	2780		2955	1991	1969	3065	3575	3182	3127	2948	3129	2948	2821	1940	3169	2964	2755	1965	2000	2836	3577	1930	3331	1931	1854	2830
EW2312-2	3031	3880	3060	3110	3058	3022	3880	3571	1571	2964	2050	3020	3020	3161	3026	2384	3161	2955		3083	3061	1878	3649	2404	2041	2248	2043	2248	2383	3032	2085	3406	3127	3058	3109	3537	3651	3021	2506	3022	2965	2719
EW2315-1	350	3559	419	918	135	594	3855	3219	3140	2000	3175	592	592	2935	598	2893	2935	1991	3083		138	3174	3626	3276	3200	2939	3202	2939	2892	351	3242	3085	2914	376	917	2945	3626	593	3455	594	774	3036
EW2321-1	328	3537	397	896	13	572	3833	3197	3116	1978	3153	570	570	2913	576	2871	2913	1969	3061	138		3152	3604	3254	3178	2917	3180	2917	2870	329	3220	3063	2892	354	895	2923	3604	571	3433	572	752	3014
EW2325-1	3122	3966	3153	3203	3149	3113	3982	3638	1805	3074	2091	3111	3111	3264	3117	2543	3264	3065	1878	3174	3152		3749	2508	2107	2411	2109	2411	2542	3123	2151	3490	3242	3149	3202	3603	3751	3112	2593	3113	3058	2770
EW2330-1	3574	4364	3603	3646	3601	3568	1795	4063	3751	3584	3770	3566	3566	3739	3572	3618	3739	3575	3649	3626	3604	3749		3809	3825	3668	3827	3668	3618	3575	3869	3893	3710	3600	3645	3718	6	3567	4092	3568	3502	3742
EW2330-2	3224	4004	3255	3307	3251	3216	4043	3621	2526	3191	2574	3214	3214	3278	3220	2540	3278	3182	2404	3276	3254	2508	3809		2619	2632	2621	2632	2539	3225	2661	3534	3252	3251	3306	3643	3811	3215	2908	3216	3157	2959
EW2335-2	3148	4090	3179	3234	3175	3138	4054	3708	2073	3136	1222	3138	3138	3387	3144	2577	3387	3127	2041	3200	3178	2107	3825	2619		2552	2	2552	2576	3149	132	3615	3361	3175	3233	3687	3827	3139	2705	3140	3087	2873
EW2338-3	2888	3757	2918	2961	2914	2878	3896	3506	2423	2957	2532	2876	2876	3079	2882	2052	3079	2948	2248	2939	2917	2411	3668	2632	2552		2554	0	2051	2889	2596	3286	3051	2914	2960	3475	3670	2877	2859	2878	2813	2829
EW2341-1	3150	4092	3181	3236	3177	3140	4056	3710	2075	3138	1224	3140	3140	3389	3146	2579	3389	3129	2043	3202	3180	2109	3827	2621	2	2554		2554	2578	3151	134	3617	3363	3177	3235	3689	3829	3141	2707	3142	3089	2875
EW2341-4	2888	3757	2918	2961	2914	2878	3896	3506	2423	2957	2532	2876	2876	3079	2882	2052	3079	2948	2248	2939	2917	2411	3668	2632	2552	0	2554		2051	2889	2596	3286	3051	2914	2960	3475	3670	2877	2859	2878	2813	2829
EW2345-3	2843	3661	2872	2926	2867	2833	3851	3407	2542	2830	2524	2831	2831	2972	2837	31	2972	2821	2383	2892	2870	2542	3618	2539	2576	2051	2578	2051		2844	2620	3185	2941	2868	2925	3360	3620	2832	2896	2833	2778	2842
EW2347-1	23	3508	370	867	326	547	3806	3170	3089	1949	3126	545	545	2884	551	2845	2884	1940	3032	351	329	3123	3575	3225	3149	2889	3151	2889	2844		3191	3034	2863	207	866	2894	3575	546	3406	547	723	2987
OKY203	3190	4132	3221	3276	3217	3180	4098	3754	2117	3178	1266	3180	3180	3429	3186	2621	3429	3169	2085	3242	3220	2151	3869	2661	132	2596	134	2596	2620	3191		3655	3399	3217	3275	3725	3871	3181	2749	3182	3129	2915
OKY98	3033	1481	3060	3099	3060	3025	4118	3465	3498	2973	3603	3023	3023	2773	3029	3186	2773	2964	3406	3085	3063	3490	3893	3534	3615	3286	3617	3286	3185	3034	3655		2749	3059	3098	3415	3893	3024	3873	3025	2952	3174
P3921	2862	3225	2889	2934	2889	2854	3932	3219	3227	2764	3320	2852	2852	967	2858	2942	967	2755	3127	2914	2892	3242	3710	3252	3361	3051	3363	3051	2941	2863	3399	2749		2888	2933	3231	3710	2853	3604	2854	2787	2890
P9252	206	3537	395	894	351	572	3830	3195	3115	1974	3150	570	570	2909	576	2869	2909	1965	3058	376	354	3149	3600	3251	3175	2914	3177	2914	2868	207	3217	3059	2888		893	2921	3600	571	3430	572	750	3012
RAC2244-1	865	3572	898	1	892	855	3880	3242	3179	2009	3212	853	853	2958	859	2926	2958	2000	3109	917	895	3202	3645	3306	3233	2960	3235	2960	2925	866	3275	3098	2933	893		2952	3645	854	3482	855	684	3053
RAC2264-1	2893	3889	2923	2953	2920	2881	3957	3571	3622	2845	3651	2879	2879	3260	2885	3361	3260	2836	3537	2945	2923	3603	3718	3643	3687	3475	3689	3475	3360	2894	3725	3415	3231	2921	2952		3718	2880	3894	2881	2804	3393
RAC2268-1	3574	4364	3603	3646	3601	3568	1797	4065	3753	3586	3772	3566	3566	3739	3572	3620	3739	3577	3651	3626	3604	3751	6	3811	3827	3670	3829	3670	3620	3575	3871	3893	3710	3600	3645	3718		3567	4094	3568	3502	3744
RAC2276-1	545	3500	574	855	568	9	3800	3154	3077	1939	3114	1	7	2874	13	2833	2874	1930	3021	593	571	3112	3567	3215	3139	2877	3141	2877	2832	546	3181	3024	2853	571	854	2880	3567		3393	3	711	2977
RAC2319-1	3405	4342	3432	3483	3430	3394	4319	3892	2557	3340	2625	3392	3392	3631	3398	2897	3631	3331	2506	3455	3433	2593	4092	2908	2705	2859	2707	2859	2896	3406	2749	3873	3604	3430	3482	3894	4094	3393		3394	3334	3045
RAC2320-2	546	3501	575	856	569	10	3801	3155	3078	1940	3115	2	8	2875	14	2834	2875	1931	3022	594	572	3113	3568	3216	3140	2878	3142	2878	2833	547	3182	3025	2854	572	855	2881	3568	3	3394		712	2978
RAC2381-2	722	3428	751	685	749	712	3734	3091	3035	1863	3066	710	710	2814	716	2779	2814	1854	2965	774	752	3058	3502	3157	3087	2813	3089	2813	2778	723	3129	2952	2787	750	684	2804	3502	711	3334	712		2911
Reference	2986	3644	3017	3054	3011	2978	3968	3421	2778	2839	2844	2976	2976	2920	2982	2841	2920	2830	2719	3036	3014	2770	3742	2959	2873	2829	2875	2829	2842	2987	2915	3174	2890	3012	3053	3393	3744	2977	3045	2978	2911	

Table S5-2 Pairwise cgSNP distance among *E. albertii* strains assigned to BAPS 2

	AKT5	AKT92	EW2206-3	EW2209-1	EW2213-2	EW2302-1	EW2335-1	EW2338-1	EW2338-2	EW2341-2	EW2341-3	EW2343-3	EW2344-2	EW2344-1	RAC2186-1	RAC2196-1	RAC2241-1	RAC2255-1	RAC2256-1	RAC2289-1	RAC2356-1	Reference
AKT5		429	7546	9835	9836	7554	5676	304	7682	7682	6939	304	6939	7755	6844	9833	9832	7758	9832	5679	7790	12555
AKT92	429		7432	9726	9727	7440	5558	283	7566	7566	6822	283	6822	7639	6733	9724	9723	7642	9723	5561	7676	12445
EW2206-3	7546	7432		9688	9689	164	7592	7402	7449	7449	7115	7402	7115	7369	6982	9686	9685	7374	9685	7595	1576	12346
EW2209-1	9835	9726	9688		1	9702	9732	9691	9569	9569	9730	9691	9730	9586	9772	4	21	9592	21	9735	9727	12550
EW2213-2	9836	9727	9689	1		9703	9733	9692	9570	9570	9731	9692	9731	9587	9773	5	22	9593	22	9736	9728	12551
EW2302-1	7554	7440	164	9702	9703		7596	7410	7449	7449	7111	7410	7111	7375	6996	9700	9699	7380	9699	7599	1568	12356
EW2335-1	5676	5558	7592	9732	9733	7596		5530	7586	7586	7298	5530	7298	7499	7082	9730	9730	7504	9730	27	7730	12446
EW2338-1	304	283	7402	9691	9692	7410	5530		7540	7540	6795	0	6795	7611	6698	9689	9688	7614	9688	5533	7646	12408
EW2338-2	7682	7566	7449	9569	9570	7449	7586	7540		0	7487	7540	7487	6268	7408	9567	9566	6275	9566	7589	7787	12245
EW2341-2	7682	7566	7449	9569	9570	7449	7586	7540	0		7487	7540	7487	6268	7408	9567	9566	6275	9566	7589	7787	12245
EW2341-3	6939	6822	7115	9730	9731	7111	7298	6795	7487	7487		6795	0	7306	5873	9728	9727	7313	9727	7301	7449	12366
EW2343-3	304	283	7402	9691	9692	7410	5530	0	7540	7540	6795		6795	7611	6698	9689	9688	7614	9688	5533	7646	12408
EW2344-2	6939	6822	7115	9730	9731	7111	7298	6795	7487	7487	0	6795		7306	5873	9728	9727	7313	9727	7301	7449	12366
EW2344-1	7755	7639	7369	9586	9587	7375	7499	7611	6268	6268	7306	7611	7306		7287	9584	9583	108	9583	7502	7639	12277
RAC2186-1	6844	6733	6982	9772	9773	6996	7082	6698	7408	7408	5873	6698	5873	7287		9770	9767	7292	9767	7085	7257	12428
RAC2196-1	9833	9724	9686	4	5	9700	9730	9689	9567	9567	9728	9689	9728	9584	9770		19	9590	19	9733	9725	12548
RAC2241-1	9832	9723	9685	21	22	9699	9730	9688	9566	9566	9727	9688	9727	9583	9767	19		9589	0	9733	9724	12547
RAC2255-1	7758	7642	7374	9592	9593	7380	7504	7614	6275	6275	7313	7614	7313	108	7292	9590	9589		9589	7507	7644	12283
RAC2256-1	9832	9723	9685	21	22	9699	9730	9688	9566	9566	9727	9688	9727	9583	9767	19	0	9589		9733	9724	12547
RAC2289-1	5679	5561	7595	9735	9736	7599	27	5533	7589	7589	7301	5533	7301	7502	7085	9733	9733	7507	9733		7733	12449
RAC2356-1	7790	7676	1576	9727	9728	1568	7730	7646	7787	7787	7449	7646	7449	7639	7257	9725	9724	7644	9724	7733		12425
Reference	12555	12445	12346	12550	12551	12356	12446	12408	12245	12245	12366	12408	12366	12277	12428	12548	12547	12283	12547	12449	12425	

Table S5-3 Pairwise cgSNP distance among *E. albertii* strains assigned to BAPS 3

	EW2204-1	EW2220-2	RAC2174-1	RAC2266-1	RAC2278-1	RAC2292-1	RAC2301-1	RAC2307-1	RAC2369-1	Reference
EW2204-1		17	23	23	19	18	1	18	21	29102
EW2220-2	17		20	20	16	15	18	13	18	29099
RAC2174-1	23	20		0	8	11	24	21	14	29099
RAC2266-1	23	20	0		8	11	24	21	14	29099
RAC2278-1	19	16	8	8		3	20	17	10	29095
RAC2292-1	18	15	11	11	3		17	14	13	29092
RAC2301-1	1	18	24	24	20	17		17	22	29101
RAC2307-1	18	13	21	21	17	14	17		19	29098
RAC2369-1	21	18	14	14	10	13	22	19		29097
Reference	29102	29099	29099	29099	29095	29092	29101	29098	29097	

Table S5-4 Pairwise cgSNP distance among *E. albertii* strains assigned to BAPS 4

	EW2206-2	EW2207-1	EW2208-1	EW2211-2	EW2213-4	EW2216-1	EW2220-1	EW2304-2	EW2305-1	EW2311-2	EW2319-1	EW2325-2	EW2325-3	EW2331-2	EW2347-2	P4660	P5051	P8234	P8514	RAC2184-1	RAC2192-1	RAC2192-2	RAC2198-2	RAC2202-1	RAC2210-1	RAC2214-2	RAC2215-1	RAC2230-1	RAC2239-1	RAC2260-1	RAC2295-2	RAC2297-1	RAC2300-1	RAC2304-1	RAC2311-1	RAC2314-1	RAC2318-1	RAC2320-1	RAC2359-2	RAC2361-1	RAC2370-1	RAC2371-1	RAC2378-1	Reference
EW2206-2		6300	6585	6134	6350	6452	6174	6405	6585	6173	6585	6329	6264	22	6444	6337	2977	2981	2981	6290	6173	6452	6584	6212	6443	6455	6461	6386	6263	6604	6611	6574	6180	6333	6402	6383	2984	6349	6327	6351	6574	6443	6585	8626
EW2207-1	6300		6516	1419	6647	6531	1192	6372	6516	1191	6516	6520	6465	6298	6434	6623	6384	6388	6388	38	1191	6531	6459	6442	6433	6429	6540	6672	6464	6480	6520	6475	1584	6524	6369	6717	6391	6646	6518	6648	6476	6433	6460	8646
EW2208-1	6585	6516		6460	6659	6627	6445	6781	0	6444	2	6503	6498	6583	6492	6642	6544	6547	6547	6507	6444	6627	6717	6528	6491	6526	6640	6691	6497	6733	6784	6933	6479	6507	6780	6736	6549	6658	6501	6660	6933	6491	6718	8784
EW2211-2	6134	1419	6460		6534	6385	713	6277	6460	712	6460	6355	6315	6132	6270	6460	6149	6153	6153	1409	712	6385	6398	6320	6269	6258	6394	6509	6314	6419	6371	6378	1628	6359	6274	6592	6156	6533	6353	6535	6379	6269	6399	8479
EW2213-4	6350	6647	6659	6534		6468	6562	6784	6659	6561	6659	6650	6580	6348	6699	6673	6314	6318	6318	6637	6561	6468	6677	6394	6698	6454	6477	6722	6579	6693	6859	6687	6594	6654	6781	6716	6319	3	6648	3	6687	6698	6678	8914
EW2216-1	6452	6531	6627	6385	6468		6403	6578	6627	6402	6627	6523	6592	6450	6572	6609	6381	6385	6385	6523	6402	0	6582	6432	6571	6591	71	6658	6591	6596	6771	6682	6452	6527	6573	6637	6388	6467	6521	6469	6682	6571	6583	8743
EW2220-1	6174	1192	6445	713	6562	6403		6299	6445	1	6445	6345	6341	6172	6286	6444	6186	6190	6190	1182	1	6403	6410	6316	6285	6282	6412	6493	6340	6431	6355	6392	1317	6349	6296	6625	6193	6561	6343	6563	6393	6285	6411	8514
EW2304-2	6405	6372	6781	6277	6784	6578	6299		6781	6298	6781	6377	6603	6403	6499	6626	6453	6457	6457	6362	6298	6578	6497	6402	6498	6586	6587	6675	6602	6511	6556	6472	6343	6381	51	6813	6460	6783	6375	6785	6473	6498	6498	8723
EW2305-1	6585	6516	0	6460	6659	6627	6445	6781		6444	2	6503	6498	6583	6492	6642	6544	6547	6547	6507	6444	6627	6717	6528	6491	6526	6640	6691	6497	6733	6784	6933	6479	6507	6780	6736	6549	6658	6501	6660	6933	6491	6718	8784
EW2311-2	6173	1191	6444	712	6561	6402	1	6298	6444		6444	6344	6340	6171	6285	6443	6185	6189	6189	1181	0	6402	6409	6315	6284	6281	6411	6492	6339	6430	6354	6391	1316	6348	6295	6624	6192	6560	6342	6562	6392	6284	6410	8513
EW2319-1	6585	6516	2	6460	6659	6627	6445	6781	2	6444		6503	6498	6583	6492	6642	6544	6547	6547	6507	6444	6627	6717	6528	6491	6526	6640	6691	6497	6733	6784	6933	6479	6507	6780	6736	6549	6658	6501	6660	6933	6491	6718	8784
EW2325-2	6329	6520	6503	6355	6650	6523	6345	6377	6503	6344	6503		6481	6327	6564	6722	6426	6430	6430	6510	6344	6523	6672	6530	6563	6538	6532	6770	6480	6684	6673	6566	6381	26	6376	6858	6433	6649	32	6651	6566	6563	6673	8748
EW2325-3	6264	6465	6498	6315	6580	6592	6341	6603	6498	6340	6498	6481		6262	6485	6737	6384	6388	6388	6455	6340	6592	6581	6508	6484	6501	6601	6786	3	6596	6707	6580	6358	6486	6600	6659	6391	6579	6480	6581	6580	6484	6582	8841
EW2331-2	22	6298	6583	6132	6348	6450	6172	6403	6583	6171	6583	6327	6262		6442	6335	2975	2979	2979	6288	6171	6450	6582	6210	6441	6453	6459	6384	6261	6602	6609	6572	6178	6331	6400	6381	2982	6347	6325	6349	6572	6441	6583	8624
EW2347-2	6444	6434	6492	6270	6699	6572	6286	6499	6492	6285	6492	6564	6485	6442		6673	6648	6652	6652	6424	6285	6572	6643	6033	1	6412	6581	6722	6484	6663	6704	6626	6313	6570	6497	6765	6655	6698	6564	6700	6626	1	6644	8660
P4660	6337	6623	6642	6460	6673	6609	6444	6626	6642	6443	6642	6722	6737	6335	6673		6387	6391	6391	6613	6443	6609	6693	6484	6672	6305	6618	121	6736	6705	6811	6651	6533	6726	6626	6721	6394	6672	6720	6674	6651	6672	6694	8900
P5051	2977	6384	6544	6149	6314	6381	6186	6453	6544	6185	6544	6426	6384	2975	6648	6387		6	6	6376	6185	6381	6644	6326	6647	6489	6390	6436	6383	6652	6766	6634	6185	6430	6452	6291	9	6313	6424	6315	6634	6647	6645	8678
P8234	2981	6388	6547	6153	6318	6385	6190	6457	6547	6189	6547	6430	6388	2979	6652	6391	6		0	6380	6189	6385	6648	6330	6651	6493	6394	6440	6387	6656	6770	6638	6189	6434	6456	6295	13	6317	6428	6319	6638	6651	6649	8682
P8514	2981	6388	6547	6153	6318	6385	6190	6457	6547	6189	6547	6430	6388	2979	6652	6391	6	0		6380	6189	6385	6648	6330	6651	6493	6394	6440	6387	6656	6770	6638	6189	6434	6456	6295	13	6317	6428	6319	6638	6651	6649	8682
RAC2184-1	6290	38	6507	1409	6637	6523	1182	6362	6507	1181	6507	6510	6455	6288	6424	6613	6376	6380	6380		1181	6523	6449	6432	6423	6419	6532	6662	6454	6470	6510	6465	1574	6514	6359	6707	6383	6636	6508	6638	6466	6423	6450	8636
RAC2192-1	6173	1191	6444	712	6561	6402	1	6298	6444	0	6444	6344	6340	6171	6285	6443	6185	6189	6189	1181		6402	6409	6315	6284	6281	6411	6492	6339	6430	6354	6391	1316	6348	6295	6624	6192	6560	6342	6562	6392	6284	6410	8513
RAC2192-2	6452	6531	6627	6385	6468	0	6403	6578	6627	6402	6627	6523	6592	6450	6572	6609	6381	6385	6385	6523	6402		6582	6432	6571	6591	71	6658	6591	6596	6771	6682	6452	6527	6573	6637	6388	6467	6521	6469	6682	6571	6583	8743
RAC2198-2	6584	6459	6717	6398	6677	6582	6410	6497	6717	6409	6717	6672	6581	6582	6643	6693	6644	6648	6648	6449	6409	6582		6512	6642	6488	6593	6742	6580	98	6736	6632	6359	6676	6495	6638	6651	6676	6668	6678	6633	6642	1	8776
RAC2202-1	6212	6442	6528	6320	6394	6432	6316	6402	6528	6315	6528	6530	6508	6210	6033	6484	6326	6330	6330	6432	6315	6432	6512		6032	6187	6441	6533	6507	6526	6613	6524	6356	6534	6398	6515	6333	6393	6530	6395	6524	6032	6513	8692
RAC2210-1	6443	6433	6491	6269	6698	6571	6285	6498	6491	6284	6491	6563	6484	6441	1	6672	6647	6651	6651	6423	6284	6571	6642	6032		6411	6580	6721	6483	6662	6703	6625	6312	6569	6496	6764	6654	6697	6563	6699	6625	0	6643	8659
RAC2214-2	6455	6429	6526	6258	6454	6591	6282	6586	6526	6281	6526	6538	6501	6453	6412	6305	6489	6493	6493	6419	6281	6591	6488	6187	6411		6600	6354	6500	6510	6620	6662	6271	6542	6583	6507	6496	6453	6536	6455	6662	6411	6489	8695
RAC2215-1	6461	6540	6640	6394	6477	71	6412	6587	6640	6411	6640	6532	6601	6459	6581	6618	6390	6394	6394	6532	6411	71	6593	6441	6580	6600		6667	6600	6607	6778	6691	6461	6536	6582	6646	6397	6476	6530	6478	6691	6580	6594	8750
RAC2230-1	6386	6672	6691	6509	6722	6658	6493	6675	6691	6492	6691	6770	6786	6384	6722	121	6436	6440	6440	6662	6492	6658	6742	6533	6721	6354	6667		6785	6754	6860	6700	6582	6774	6675	6770	6443	6721	6768	6723	6700	6721	6743	8949
RAC2239-1	6263	6464	6497	6314	6579	6591	6340	6602	6497	6339	6497	6480	3	6261	6484	6736	6383	6387	6387	6454	6339	6591	6580	6507	6483	6500	6600	6785		6595	6706	6579	6357	6485	6599	6658	6390	6578	6479	6580	6579	6483	6581	8840
RAC2260-1	6604	6480	6733	6419	6693	6596	6431	6511	6733	6430	6733	6684	6596	6602	6663	6705	6652	6656	6656	6470	6430	6596	98	6526	6662	6510	6607	6754	6595		6748	6646	6381	6688	6509	6644	6659	6692	6682	6694	6647	6662	99	8792
RAC2295-2	6611	6520	6784	6371	6859	6771	6355	6556	6784	6354	6784	6673	6707	6609	6704	6811	6766	6770	6770	6510	6354	6771	6736	6613	6703	6620	6778	6860	6706	6748		6717	6379	6677	6555	6938	6773	6858	6671	6860	6719	6703	6737	8834
RAC2297-1	6574	6475	6933	6378	6687	6682	6392	6472	6933	6391	6933	6566	6580	6572	6626	6651	6634	6638	6638	6465	6391	6682	6632	6524	6625	6662	6691	6700	6579	6646	6717		6379	6570	6472	6836	6641	6686	6564	6688	30	6625	6633	8962
RAC2300-1	6180	1584	6479	1628	6594	6452	1317	6343	6479	1316	6479	6381	6358	6178	6313	6533	6185	6189	6189	1574	1316	6452	6359	6356	6312	6271	6461	6582	6357	6381	6379	6379		6385	6340	6618	6192	6593	6379	6595	6380	6312	6360	8543
RAC2304-1	6333	6524	6507	6359	6654	6527	6349	6381	6507	6348	6507	26	6486	6331	6570	6726	6430	6434	6434	6514	6348	6527	6676	6534	6569	6542	6536	6774	6485	6688	6677	6570	6385		6380	6862	6437	6653	36	6655	6570	6569	6677	8752
RAC2311-1	6402	6369	6780	6274	6781	6573	6296	51	6780	6295	6780	6376	6600	6400	6497	6626	6452	6456	6456	6359	6295	6573	6495	6398	6496	6583	6582	6675	6599	6509	6555	6472	6340	6380		6808	6459	6780	6374	6782	6473	6496	6496	8719
RAC2314-1	6383	6717	6736	6592	6716	6637	6625	6813	6736	6624	6736	6858	6659	6381	6765	6721	6291	6295	6295	6707	6624	6637	6638	6515	6764	6507	6646	6770	6658	6644	6938	6836	6618	6862	6808		6296	6715	6856	6717	6836	6764	6639	8959
RAC2318-1	2984	6391	6549	6156	6319	6388	6193	6460	6549	6192	6549	6433	6391	2982	6655	6394	9	13	13	6383	6192	6388	6651	6333	6654	6496	6397	6443	6390	6659	6773	6641	6192	6437	6459	6296		6318	6431	6320	6641	6654	6652	8685
RAC2320-1	6349	6646	6658	6533	3	6467	6561	6783	6658	6560	6658	6649	6579	6347	6698	6672	6313	6317	6317	6636	6560	6467	6676	6393	6697	6453	6476	6721	6578	6692	6858	6686	6593	6653	6780	6715	6318		6647	4	6686	6697	6677	8913
RAC2359-2	6327	6518	6501	6353	6648	6521	6343	6375	6501	6342	6501	32	6480	6325	6564	6720	6424	6428	6428	6508	6342	6521	6668	6530	6563	6536	6530	6768	6479	6682	6671	6564	6379	36	6374	6856	6431	6647		6649	6564	6563	6669	8746
RAC2361-1	6351	6648	6660	6535	3	6469	6563	6785	6660	6562	6660	6651	6581	6349	6700	6674	6315	6319	6319	6638	6562	6469	6678	6395	6699	6455	6478	6723	6580	6694	6860	6688	6595	6655	6782	6717	6320	4	6649		6688	6699	6679	8915
RAC2370-1	6574	6476	6933	6379	6687	6682	6393	6473	6933	6392	6933	6566	6580	6572	6626	6651	6634	6638	6638	6466	6392	6682	6633	6524	6625	6662	6691	6700	6579	6647	6719	30	6380	6570	6473	6836	6641	6686	6564	6688		6625	6634	8962
RAC2371-1	6443	6433	6491	6269	6698	6571	6285	6498	6491	6284	6491	6563	6484	6441	1	6672	6647	6651	6651	6423	6284	6571	6642	6032	0	6411	6580	6721	6483	6662	6703	6625	6312	6569	6496	6764	6654	6697	6563	6699	6625		6643	8659
RAC2378-1	6585	6460	6718	6399	6678	6583	6411	6498	6718	6410	6718	6673	6582	6583	6644	6694	6645	6649	6649	6450	6410	6583	1	6513	6643	6489	6594	6743	6581	99	6737	6633	6360	6677	6496	6639	6652	6677	6669	6679	6634	6643		8777
Reference	8626	8646	8784	8479	8914	8743	8514	8723	8784	8513	8784	8748	8841	8624	8660	8900	8678	8682	8682	8636	8513	8743	8776	8692	8659	8695	8750	8949	8840	8792	8834	8962	8543	8752	8719	8959	8685	8913	8746	8915	8962	8659	8777	

Table S5-5 Pairwise cgSNP distance among *E. albertii* strains assigned to BAPS 5

	EW2210-1	EW2312-1	RAC2179-1	RAC2182-1	RAC2198-1	RAC2199-1	RAC2214-1	RAC2277-1	RAC2354-1	RAC2368-1	Reference
EW2210-1		11	4	13206	13206	13215	13203	13215	13208	13205	27359
EW2312-1	11		13	13201	13201	13210	13198	13210	13203	13200	27354
RAC2179-1	4	13		13208	13208	13217	13205	13217	13210	13207	27361
RAC2182-1	13206	13201	13208		46	55	43	55	22	9	27239
RAC2198-1	13206	13201	13208	46		55	41	55	48	45	27238
RAC2199-1	13215	13210	13217	55	55		52	4	57	54	27246
RAC2214-1	13203	13198	13205	43	41	52		52	45	42	27234
RAC2277-1	13215	13210	13217	55	55	4	52		57	54	27246
RAC2354-1	13208	13203	13210	22	48	57	45	57		21	27239
RAC2368-1	13205	13200	13207	9	45	54	42	54	21		27238
Reference	27359	27354	27361	27239	27238	27246	27234	27246	27239	27238	

Table S5-6 Pairwise cgSNP distance among *E. albertii* strains assigned to BAPS 6

	EW2304-1	RAC2177-1	RAC2182-2	RAC2248-1	RAC2359-1	RAC2381-1	Reference
EW2304-1		10	8	12	10	7	28708
RAC2177-1	10		4	6	4	5	28706
RAC2182-2	8	4		8	6	3	28702
RAC2248-1	12	6	8		4	7	28708
RAC2359-1	10	4	6	4		5	28706
RAC2381-1	7	5	3	7	5		28703
Reference	28708	28706	28702	28708	28706	28703	

Table S6 Genetic characteristics of *E. albertii* strains selected from environmental water and raccoon sources.

Strain	Sampling date	SNP distance	Sampling location	EAOg	cgST[a]	Virulence genes other than *Eacdt*	Plasmid typing[b]
EW2338-3	2023/7/31	0	Kaizuka city	5	178672	*paa* , *eae*	-
EW2341-4	2023/8/7		Kaizuka city	5	178672	*paa* , *eae*	-
EW2216-2	2022/12/3	31	Izumisano city	UT	178672	*paa* , *eae*	IncFII
EW2345-3	2023/9/4		Kaizuka city	UT	178672	*paa* , *eae* , *Eccdt-I*	IncFII, IncI(Gamma)
EW2335-2	2023/7/3	2	Kaizuka city	UT	142064	*paa* , *eae*	IncFII
EW2341-1	2023/8/7		Kaizuka city	UT	142064	*paa* , *eae*	IncFII
EW2338-2	2023/7/31	0	Kaizuka city	UT	28841	*paa* , *eae*	-
EW2341-2	2023/8/7		Kaizuka city	UT	28841	*paa* , *eae*	-
EW2338-1	2023/7/31	0	Kaizuka city	18	155328	*paa* , *eae*	IncFII
EW2343-3	2023/8/7		Kaizuka city	18	155328	*paa* , *eae*	IncFII
EW2341-3	2023/8/7	0	Kaizuka city	UT	171831	*paa* , *eae*	-
EW2344-2	2023/9/4		Kaizuka city	UT	171831	*paa* , *eae*	IncFIB(AP001918)
EW2330-1	2023/5/12	6	Izumisano city	39	64189	*paa* , *eae* , *Eccdt-I*	-
RAC2268-1	2022/7/11		Tondabayashi city	39	64189	*paa* , *eae* , *Eccdt-I*	-
EW2213-3	2022/10/17	0	Hannan city	40	136945	*paa* , *eae* , *Eccdt-I*	IncFIC(FII), IncI2(Delta), pO111
EW2302-2	2023/1/9		Hannan city	40	136945	*paa* , *eae* , *Eccdt-I*	IncFIC(FII), pO111
EW2202-1	2022/8/16	1	Izumisano city	29	3327	*Eapaa* , *eae* , *Eccdt-I* , *stx2f*	IncFIB(AP001918), pO111
RAC2244-1	2022/6/1		Kaizuka city	29	3327	*Eapaa* , *eae* , *Eccdt-I* , *stx2f*	IncFIB(AP001918), pO111
EW2213-1	2022/10/17	1–14	Hannan city	UT	130744	*paa* , *eae* , *Eccdt-I*	IncFIB(AP001918), IncY
EW2212-1	2022/10/17		Izumisano city	UT	130744	*paa* , *eae* , *Eccdt-I*	IncFIB(AP001918)
EW2205-1	2022/9/13		Izumisano city	UT	130744	*paa* , *eae* , *Eccdt-I*	IncFIB(AP001918), Col(pHAD28)
EW2211-1	2022/10/3		Izumisano city	UT	130744	*paa* , *eae* , *Eccdt-I*	IncFIB(AP001918)
RAC2276-1	2022/8/8		Kaizuka city	UT	130744	*paa* , *eae* , *Eccdt-I*	IncFIB(AP001918)
RAC2320-2	2022/10/19		Hannan city	UT	147747	*paa* , *eae* , *Eccdt-I*	IncFIB(AP001918), IncFIC(FII)
EW2347-1	2023/10/7	23	Izumisano city	11	105336	*paa* , *eae* , *Eccdt-I*	IncFII, IncI(Gamma)
17002			Tochigi prefecture	11	105336	*paa* , *eae* , *Eccdt-I*	IncFIB(AP001918)
EW2321-1	2023/3/21	13	Higashiosaka city	11	105336	*paa* , *eae* , *Eccdt-I*	IncFIB(AP001918)
EW2204-2	2022/9/5		Izumisano city	11	105336	*paa* , *eae* , *Eccdt-I*	IncFIB(AP001918)
RAC2256-1	2022/6/13	1–21	Kaizuka city	19	105331	*paa* , *eae*	IncFIB(AP001918), IncFII(pHN7A8), Col156
RAC2241-1	2022/6/1		Kawachinagano city	19	105331	*paa* , *eae*	IncFIB(AP001918), IncFII(pHN7A8)
RAC2196-1	2022/2/22		Hirakata city	19	105331	*paa* , *eae*	IncFIB(AP001918), IncFII(pHN7A8)

EW2209-1	2022/10/3		Kaizuka city	19	105331	*paa* , *eae*	IncFIB(AP001918), IncFII(pHN7A8)
EW2213-2	2022/10/17		Hannan city	19	105331	*paa* , *eae*	IncFIB(AP001918), IncFII(pHN7A8)
RAC2177-1	2021/11/30	3–12	Hannan city	10	2379	*paa* , *eae*	ColpVC
RAC2381-1	2023/2/1		Kawachinagano city	10	2379	*paa* , *eae*	-
RAC2248-1	2022/6/8		Kawachinagano city	10	2379	*paa* , *eae*	-
RAC2182-2	2021/12/6		Hannan city	10	2379	*paa* , *eae*	-
RAC2359-1	2022/11/29		Settsu city	10	2379	*paa* , *eae*	-
EW2304-1	2023/1/9		Sennan city	10	2379	*paa* , *eae*	-
RAC2289-1	2022/9/5	27	Katano city	18	92242	*paa* , *eae*	IncFIB(AP001918), IncFII(pHN7A8), Col156, ColpVC
EW2335-1	2023/7/3		Kaizuka city	18	92242	*paa* , *eae*	IncFII
EW2207-1	2022/9/27		Izumisano city	25	138216	*paa* , *eae*	-
RAC2184-1	2021/12/14		Kawachinagano city	25	138216	*paa* , *eae*	-
EW2220-1	2022/12/17	1	Izumisano city	25	138216	*paa* , *eae*	IncFIB(AP001918)
EW2311-2	2023/2/15		Sennan city	25	138216	*paa* , *eae*	IncFIB(AP001918)
RAC2192-1	2022/2/1		Hannan city	25	138216	*paa* , *eae*	IncFIB(AP001918)
EW2206-2	2022/9/13		Izumisano city	31	1701	*paa* , *eae*	pO111
EW2331-2	2023/5/12		Izumisano city	31	1701	*paa* , *eae*	-
RAC2318-1	2022/10/19	6–9	Kawachinagano city	1	2370	*paa* , *eae*	-
P8234			Okayama prefecture	1	2370	*paa* , *eae*	-
P8514			Okayama prefecture	1	2370	*paa* , *eae*	-
P5051			Okayama prefecture	1	2370	*paa* , *eae*	-
EW2347-2	2023/10/7	1	Izumisano city	UT	159786	*paa* , *eae*	-
RAC2371-1	2022/12/14		Kaizuka city	UT	159786	*paa* , *eae*	IncFIB(AP001918), IncFIC(FII)
RAC2210-1	2022/4/6		Kaizuka city	UT	159786	*paa* , *eae*	-
RAC2297-1	2022/9/5		Hannan city	9	118707	*paa* , *eae*	-
RAC2370-1	2022/12/14		Kawachinagano city	9	118707	*paa* , *eae*	-
RAC2369-1	2022/12/6	1–23	Kaizuka city	5	159882	*paa* , *eae*	IncFIB(AP001918)
RAC2266-1	2022/7/11		Kawachinagano city	5	159882	*paa* , *eae*	-
RAC2174-1	2021/11/16		Kawachinagano city	5	159882	*paa* , *eae*	-
RAC2292-1	2022/9/5		Hirakata city	5	159882	*paa* , *eae*	-
RAC2278-1	2022/8/15		Hirakata city	5	159882	*paa* , *eae*	-
RAC2307-1	2022/10/5		Kawachinagano city	5	159882	*paa* , *eae*	-
RAC2301-1	2022/9/12		Kaizuka city	5	159882	*paa* , *eae*	Col156, ColpVC, IncFII

EW2220-2	2022/12/17		Izumisano city	5	159882	*paa* , *eae*	-
EW2204-1	2022/9/5		Izumisano city	5	159882	*paa* , *eae*	-
RAC2192-2	2022/2/1	0	Hannan city	UT	6934	*paa* , *eae*	-
EW2216-1	2022/12/3		Izumisano city	UT	6934	*paa* , *eae*	-
RAC2198-2	2022/2/22	1	Kawachinagano city	34	2378	*paa* , *eae*	IncFIB(AP001918), IncFIC(FII)
RAC2378-1	2023/1/18		Kawachinagano city	34	2378	*paa* , *eae*	IncFIB(AP001918), IncFIB(H89-PhagePlasmid), IncFIC(FII)
EW2213-4	2022/10/17	3–4	Hannan city	UT	147747	*paa* , *eae*	IncFIB(AP001918), IncFIC(FII)
RAC2361-1	2022/11/29		Tondabayashi city	UT	14932	*paa* , *eae*	IncFIB(AP001918), IncFIC(FII)
RAC2320-1	2022/10/19		Hannan city	UT	147747	*paa* , *eae*	IncFIB(AP001918), IncFIC(FII)
RAC2311-1	2022/10/13	51	Kawachinagano city	UT	138216	*paa* , *eae*	-
EW2304-2	2023/1/9		Sennan city	UT	138216	*paa* , *eae*	IncFIB(AP001918), IncFIC(FII)
EW2325-2	2023/4/24	26–32	Hannan city	25	159889	*paa* , *eae*	-
RAC2304-1	2022/9/27		Tondabayashi city	25	159889	*paa* , *eae*	-
RAC2359-2	2022/11/29		Settsu city	25	159889	*paa* , *eae*	-
EW2325-3	2023/4/24	3	Hannan city	7	2379	*paa* , *eae*	IncFIB(AP001918), IncFII
RAC2239-1	2022/6/1		Izumisano city	7	2379	*paa* , *eae*	IncFIB(AP001918), IncFII
EW2319-1	2023/3/21	0–2	Kanan town	UT	159889	*paa* , *eae*	IncFIB(AP001918)
EW2305-1	2023/1/17		Kaizuka city	UT	159889	*paa* , *eae*	IncFIB(AP001918)
EW2208-1	2022/9/27		Izumisano city	UT	159889	*paa* , *eae*	IncFIB(AP001918)
RAC2179-1	2021/12/6	4–13	Izumisano city	16	2384	*paa* , *eae*	IncFIB(AP001918), IncFII, IncI1-I(Alpha)
EW2312-1	2023/2/15		Sennan city	16	2384	*paa* , *eae*	IncFIB(AP001918), IncFIC(FII)
EW2210-1	2022/10/3		Kaizuka city	16	2384	*paa* , *eae*	IncFIB(AP001918), IncFII
RAC2368-1	2022/12/6	4–55	Tajiri town	21	2384	*paa* , *eae*	IncFIB(AP001918), IncFII
RAC2354-1	2022/11/24		Tondabayashi city	21	2384	*paa* , *eae*	IncFIB(AP001918), IncFII
RAC2182-1	2021/12/6		Hannan city	21	2384	*paa* , *eae*	Col156
RAC2198-1	2022/2/22		Kawachinagano city	21	2384	*paa* , *eae*	IncFIB(AP001918), IncFII
RAC2214-1	2022/4/20		Fujiidera city	21	2384	*paa* , *eae*	IncFIB(AP001918), IncFII
RAC2277-1	2022/8/8		Kaizuka city	21	2384	*paa* , *eae*	IncFIB(AP001918), IncFII
RAC2199-1	2022/3/2		Izumisano city	21	2384	*paa* , *eae*	IncFIB(AP001918), IncFII, IncX1

[a]cgST was identified from draft genome of each strain using cgMLSTFinder (v1.2) with *E. coli* database. -, no whole genome sequencing was done.

[b]Plasmid detection and typing was done using Plasmid Finder 2.1 with the Enterobacteriales database (https://cge.food.dtu.dk/services/PlasmidFinder/). '-' indicates no plasmid detected.

Table S7. Detailed information of clinical *E. albertii* strains included in this study.

Strain	EAOg	rhierBAPS	Source	Reference
17002	11	1	Outbreak in Tochigi, Japan in 2017	1
P3921	40	1	Okayama, Japan	2
P4660	18	4	Okayama, Japan	2
P5051	1	4	Okayama, Japan	2
P8234	1	4	Okayama, Japan	2
P8514	1	4	Okayama, Japan	2
P9252	11	1	Okayama, Japan	This study
OKY98	8	1	Okayama, Japan	3
OKY203	UT	1	Okayama, Japan	3
AKT5	18	2	Akita Japan	4
AKT92	18	2	Akita Japan	4

References

1. Ishioka M, Seki A, Nakata Y, T. T, Wakatsuki A, Kataoka S, Tokoi Y. 2016. Foodborne outbreak caused by *Escherichia albertii* in Utsunomiya city [in Japanese]. Infect Agents Surveill Rep 37:98-100.

2. Hinenoya A, Yasuda N, Mukaizawa N, Sheikh S, Niwa Y, Awasthi SP, Asakura M, Tsukamoto T, Nagita A, Albert MJ, Yamasaki S. 2017. Association of cytolethal distending toxin-II gene-positive *Escherichia coli* with *Escherichia albertii*, an emerging enteropathogen. Int J Med Microbiol 307:564-571.

3. Awasthi SP, Nagita A, Hatanaka N, Hassan J, Xu B, Hinenoya A, Yamasaki S. 2024. Detection of prolong excretion of *Escherichia albertii* in stool specimens of a 7-year-old child by a newly developed *Eacdt* gene-based quantitative real-time PCR method and molecular characterization of the isolates. Heliyon 10:e30042.

4. Hinenoya A, Ichimura H, Awasthi SP, Yasuda N, Yatsuyanagi J, Yamasaki S. 2019. Phenotypic and molecular characterization of *Escherichia albertii*: Further surrogates to avoid potential laboratory misidentification. Int J Med Microbiol 309:108-115.
